# *In-vitro* evaluation of synthetic dye decolourisation by filamentous ascomycetous fungi isolated from freshwater environments in Sri Lanka and development of a prototype for addressing environmental pollution from synthetic dye contamination

**DOI:** 10.3389/fcimb.2025.1650835

**Published:** 2025-10-23

**Authors:** Madhara K. Wimalasena, Nalin N. Wijayawardene, Susan B. Dharmarathne, Nimesha M. Gunasekara, Manavi S. Ekanayake, R. G. Udeni Jayalal, Jayarama D. Bhat, Turki M. Dawoud, Gui-Qing Zhang, Damith C. Weerasinghe, Heethaka K. S. de Zoysa, Dong-Qin Dai, Haohan Wang, Thushara C. Bamunuarachchige

**Affiliations:** ^1^ Center for Yunnan Plateau Biological Resources Protection and Utilization & Yunnan International Joint Laboratory of Fungal Sustainable Utilization in South and Southeast Asia, College of Biology and Food Engineering, Qujing Normal University, Qujing, China; ^2^ Department of Bioprocess Technology, Faculty of Technology, Rajarata University of Sri Lanka, Mihintale, Sri Lanka; ^3^ Faculty of Graduate Studies, Sabaragamuwa University of Sri Lanka, Belihuloya, Sri Lanka; ^4^ Tropical Microbiology Research Foundation, Pannipitiya, Sri Lanka; ^5^ Department of Aquaculture and Fisheries, Faculty of Livestock, Fisheries and Nutrition, Wayamba University of Sri Lanka, Makandura, Sri Lanka; ^6^ Department of Natural Resources, Faculty of Applied Sciences, Sabaragamuwa University of Sri Lanka, Belihuloya, Sri Lanka; ^7^ Department of Botany and Microbiology, College of Science, King Saud University, Riyadh, Saudi Arabia; ^8^ Biology Division, Vishnugupta Vishwavidyapeetam, Ashoke, Gokarna, India; ^9^ Formerly, Department of Botany, Goa University, Goa, India; ^10^ Department of Biology, Faculty of Science, Chiang Mai University, Chiang Mai, Thailand; ^11^ Center of Excellence in Microbial Diversity and Sustainable Utilization, Chiang Mai University, Chiang Mai, Thailand; ^12^ Department of Materials Technology, Faculty of Technology, Rajarata University of Sri Lanka, Mihintale, Sri Lanka

**Keywords:** enzymatic activity, laboratory dyes, *Lasiodiplodia pseudotheobromae*, mycoremediation, microencapsulation system

## Abstract

Mycoremediation emerges as an alternative strategy for decolourisation of synthetic dyes and is valued for its cost-effectiveness and environmentally friendly attributes. Five fungal strains, *Aspergillus* sp.1, *Lasiodiplodia crassispora*, *L. pseudotheobromae*, *Neopestalotiopsis saprophytica*, and *Trichoderma* sp.1, isolated from freshwater environments in Sri Lanka, were subjected to decolourisation of 100 mg L^-1^ of Congo Red (CR), Crystal Violet (CV), Malachite Green (MG), and Safranin dyes, frequently discharged into the environment from laboratories and industries. Screening of the decolourisation ability of isolated fungal strains was conducted in both solid and liquid media containing Potato Dextrose Agar (PDA) and Potato Dextrose Broth (PDB) for ten days incubation period and 14–28 days, respectively, at 30 °C. The liquid media screening processes showed that *L. pseudotheobromae* exhibited the highest decolourisation percentage for CV (95.23% ± 0.82) and MG (93.12% ± 0.36). *L. crassispora* demonstrated the highest decolourisation abilities for CR (91.45% ± 0.20) and Safranin. All fungal strains successfully achieved over 60% decolourisation of CV, CR, and MG. However, Safranin showed the lowest decolourisation by all isolated strains, except for *L. crassispora* (70.46% ± 1.18). Considering the overall results in both solid and liquid media (exceeding 70%), *L. crassispora* exhibited the highest decolourisation ability among all selected dyes. Besides, the results in liquid media were reconfirmed by the screening process on solid media. The results of the present study showed that mycoremediation for synthetic dye decolourisation should be expanded to outdoor settings. Leveraging this insight, a prototype was developed for real-world application, creating a microencapsulation system for mycoremediation. This innovative system offers a sustainable alternative to traditional physicochemical treatments for wastewater management, specifically on laboratory discharges.

## 1 Introduction

Widespread application of synthetic dyes in diverse industries, including paints, textiles, papers, cosmetics, tannery products, food, and pharmaceuticals, leads to the discharge of substantial amounts of coloured and toxic effluents into water sources (Palapa et al., 2021; Slama et al., 2021; Ganaie et al., 2023). Approximately 280,000 tons of dyes are discharged annually through industrial wastewater (Gomaa et al., 2023; Xing et al., 2024). The direct discharge of synthetic dye-contaminants into aquatic systems can adversely affect both environmental and human aspects (Ardila-Leal et al., 2021; Li et al., 2021; Bharathi and Ga, 2022). Synthetic dye contaminants interfere with gas solubility and light penetration in water, thereby curtailing photosynthesis in aquatic ecosystems, destroying food chains, disrupting growth in aquatic biota, and also posing health risks due to their cancerous and mutagenic properties (Chen et al., 2019; Gao et al., 2020; Li et al., 2021; Rápó and Tonk, 2021; Sudarshan et al., 2023).

Synthetic dyes exhibited notable resistance against natural degradation mechanisms due to their intricate chemical structures (Ekanayake and Manage, 2022; Rodrigues et al., 2023). Chemical, physical, and hybrid approaches are extensively employed for colour removal from wastewater sludges (Rápó and Tonk, 2021; Ganaie et al., 2023; Khan et al., 2023). Prevalent physical techniques consist of adsorption, membrane separation, and ion exchange, while chemical methods encompass electrochemical processes, advanced oxidation, coagulation, and flocculation, widely utilised in synthetic dyes decolourisation (Rápó and Tonk, 2021; Srivastava et al., 2022; Ganaie et al., 2023; Khan et al., 2023; Thoa et al., 2023). However, physicochemical treatments have disadvantages in producing sludge, leading to management difficulties, high costs, and requiring extensive treatment areas (Yang et al., 2016; Routoula and Patwardhan, 2020; Ardila-Leal et al., 2021; Shah et al., 2023).

Mycoremediation serves as an alternative strategy for decolourising synthetic dyes and is recognised for its cost-effectiveness and environmentally friendly characteristics in removing colour contaminants from wastewater (Yang et al., 2016; Chen et al., 2019; Melati et al., 2022; Thoa et al., 2023). Fungi have a distinctive capability to metabolize various pollutant compounds and utilize them as energy and carbon sources by breaking them down into less toxic forms (Shourie and Vijayalakshmi, 2022; Vaksmaa et al., 2023). In addition, fungi possess the ability to produce extracellular degradative enzymes, such as manganese peroxidase (MnP), lignin peroxidase (LiP), and laccase (Lac) (Ekanayake and Manage, 2022; Kumar and Arora, 2022; Saikia et al., 2023). Biodegradation and biosorption play crucial roles in the fungal decolourisation process and are facilitated by the function of extracellular enzymes (Yang et al., 2016; Bibbins-Martínez et al., 2023). The oxidation reactions of these enzymes facilitate the degradation of intricate pollutant structures into metabolite intermediates that are more readily degradable (Vaksmaa et al., 2023). These enzymatic activities efficiently decolourize synthetic dyes in wastewater (Eichlerová and Baldrian, 2020; Gao et al., 2020; Shah et al., 2023).

In order to control the severe water pollution resulting from improper application of synthetic dyes and their discharge without adequate treatment in our current environment, it is essential to pursue sustainable alternatives (Slama et al., 2021; Akter et al., 2023; Alzain et al., 2023; Intisar et al., 2023), especially by conducting a thorough assessment of fungi and to identify those that can efficiently neutralize the dye from wastewater.

The primary objective of this research is to move beyond laboratory-scale evaluations of synthetic dye decolourising fungi and advance towards real-world application by developing a prototype system for the treatment of synthetic dye contaminated wastewater. Besides, we wanted to emphasise that common species can also be utilised in different industrial aspects as well. Consequently, this study investigates the potential of filamentous ascomycetous fungi isolated from freshwater environments in Sri Lanka by evaluating their ability to decolourise selected synthetic dyes that are frequently discharged into the environment from laboratories and industries. Accordingly, *Aspergillus* sp., *Lasiodiplodia crassispora, L. pseudotheobromae, Neopestalotiopsis saprophytica*, and *Trichoderma* sp. were selected as representative filamentous ascomycetous fungi, while Congo Red (CR), Crystal Violet (CV), Malachite Green (MG), and Safranin were chosen as the synthetic dyes for this study. These synthetic dyes, recognised as toxic substances, are commonly used in laboratory experiments (Islam et al., 2019; Bharathi and Ga, 2022; Shah et al., 2023). Based on solid and liquid media assays, the best-performing fungus (*L. pseudotheobromae*) for synthetic dye decolourisation and the most effectively decolourised dye (CV) were selected to develop a prototype that represents the first and second stages of prototype development.

## 2 Methodology

### 2.1 Dyes and media

Considering the laboratory dye usage, four structurally different synthetic dyes; one azo dye - CR, phenazine dye - Safranin, and two Triphenylmethane dyes - CV and MG were obtained from Himedia Laboratories Pvt. Ltd., Mumbai, India, and used for primary mycoremediation screenings. All the synthetic dyes were of analytical grade.

Water Agar medium (WA; 1.5% agar) was employed for isolation of fungi (Yang et al., 2016), while potato dextrose agar medium (PDA; 20% potato, 2% dextrose, 2% agar) was used for fungal colony growth, and as a solid medium for synthetic dye decolourisation experiment (Jayasinghe et al., 2008; Rani et al., 2014; Ekanayake and Manage, 2022). Potato Dextrose Broth (PDB; 20% potato, 2% dextrose) was used for the decolourisation test in a liquid medium (Jayasinghe et al., 2008; Purnomo et al., 2019).

### 2.2 Isolation of fungal strains

**Figure 1:**
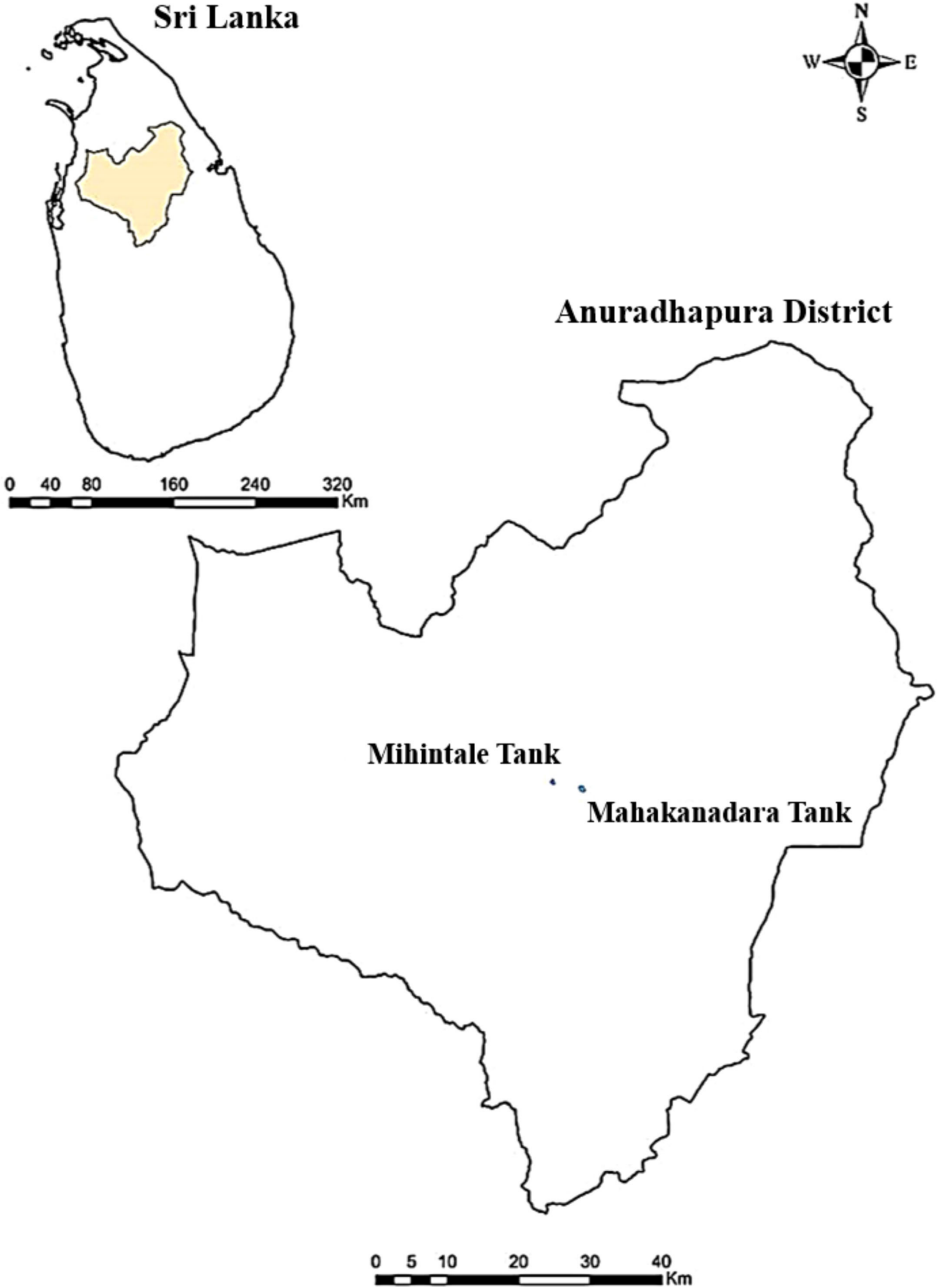
Locations where specimens were collected in the Anuradhapura district, Sri Lanka.

Submerged dead woody specimens with fungal fruiting bodies were collected from lentic freshwater habitats (Mahakanadara tank; 8.38683° N, 80.38683° E, and Mihintale tank; 8.36267° N, 80.50591° E) in Anuradhapura district, Sri Lanka (**Figure 1**). The specimens were brought to the laboratory in Ziplock plastic bags and incubated using moist chambers at room temperature for three days. Pure cultures were obtained following the single spore isolation technique described by Chomnunti et al. (2014) and Senanayake et al. (2020) on PDA media under aseptic conditions at room temperature (28 °C – 30 °C) for three to five days, and stock cultures were maintained at 4 °C. Living cultures were deposited at the Rajarata University Fungal Culture Collection (RUFCC) in Sri Lanka.

### 2.3 Colony and morphological characterization

Macro and micro-morphological characters were observed using a stereomicroscope (Nikon SMZ18; Nikon Corporation Shinagawa Intercity Tower C, 2-15-3, Konan, Minato-ku, Tokyo, 108–6290 Japan) and an inverted trinocular microscope (Nikon ECLIPSE Ts2R-FL; Nikon Corporation Shinagawa Intercity Tower C, 2-15-3, Konan, Minato-ku, Tokyo, 108–6290 Japan), respectively. Colony characteristics (colour, form, elevation, margin, and texture) were examined and recorded over a period ranging from three to seven days, and images were subsequently processed using the extended version of the online photo editor Photopea (https://www.photopea.com/; accessed on 10 January 2024).

### 2.4 Molecular identification

#### 2.4.1 Genomic DNA extraction

Genomic DNA was extracted from fresh fungal mycelium grown on PDA at room temperature (~28 °C) under ambient light for one week. The extraction process followed the Cetyltrimethylammonium Bromide (CTAB) method, adhering to the protocols of Gontia-Mishra et al. (2014). Accordingly, Fresh fungal mycelium (~300 mg) was transferred into a sterilised 1.5 mL microcentrifuge tube with 800 µL of CTAB extraction buffer (0.1 M Tris-HCl pH 8, 10 mM EDTA pH 8, 2.5 M NaCl, 3.5% CTAB, and 150 µL of 20 mg/mL proteinase K), along with sterilised 0.5–1 mm glass beads. The mixture was vortexed for 5 min. and incubated at 65 °C for 30 min. After centrifugation at 12,000 rpm for 10 min., the supernatant was extracted and mixed with phenol-chloroform-isoamyl alcohol (25:24:1), followed by another round of centrifugation. The upper phase was again treated with chloroform-isoamyl alcohol (24:1), centrifuged, and the supernatant was precipitated with ice-cold isopropanol. DNA was pelleted by centrifugation at 13,000 rpm for 15 min., washed with 70% ethanol, air-dried, and dissolved in 50 µL TE buffer.

#### 2.4.2 PCR amplification and estimation of fragment size

PCR amplification and sequencing were conducted for all species in the internal transcribed spacers (ITS) nrDNA region using the primer pair ITS4 (White et al., 1990) and ITS1F (Gardes and Bruns, 1993) as previously described by White et al. (1990). Due to their high performance in synthetic dye decolourisation, the identification of *Lasiodiplodia crassispora* and *L. pseudotheobromae* species was further confirmed through molecular identification using the Small-subunit ribosomal RNA (SSU; NS1/NS4, White et al., 1990) and Translation Elongation Factor 1 (*tef*1-α; EF1-728F/EF1-986R, Carbone and Kohn, 1999) gene regions.

The final volume of the PCR reaction was 25 μl, containing 5 μl of DNA template, 2.5 μl of each forward and reward primer, 12.5 μl of HIMEDI A MBT061-100R 2× PCR TaqMixture (mixture of Taq DNA Polymerase, dNTPs, and optimised buffer) and 2.5 μl of ddH_2_O. The PCR amplification was performed with an initial denaturing step of 95 °C for 5 min., followed by 40 amplification cycles in a denaturation step of 95 °C for 1 min., an extension step of 72 °C for 1 min., and a final extension step of 72 °C for 10 min. The annealing temperatures were set for the gene loci, with the optimum for each: SSU: 55 °C, ITS and *tef*1-α: 54 °C for 5 min. All PCR products were visualised by 1% agarose gel (stained with Diamond TM Nucleic Acid Dye) electrophoresis at 60 V/cm for 30 min. The gel was visualised under a UV transilluminator to estimate the fragment size.

#### 2.4.3 DNA sequencing

PCR products were sequenced using Next-Generation Sequencing (NGS) and Sanger sequencing (bidirectional). The obtained sequence was compared with entries in the GenBank database to identify significant alignments with 100% similarity using the Basic Local Alignment Search Tool (BLAST) (https://blast.ncbi.nlm.nih.gov; accessed on 03 January 2024).

#### 2.4.4 Phylogenetic analysis

Based on ITS and *tef*1-α gene blast similarity, and related publications, the closely related sequences were downloaded from GenBank (**Table 1**). ITS and *tef*1-α gene sequences were aligned by the online tool Mafft v.7.215 (http://mafft.cbrc.jp/alignment/server/index.html) (*fide* Katoh and Standley, 2013). BioEdit v.7.0.5.2 was performed to improve and combine the ITS and *tef*1-α gene alignment (Hall, 2004). The online tool ALTER (http://sing.ei.uvigo.es/ALTER/) was carried out to convert the combined datasets in FASTA format to PHYLIP and NEXUA formats (Glez-Peña et al., 2010). Maximum Likelihood (ML) analysis was presented by using the online portal CIPRES Science Gateway v. 3.3 (Miller et al., 2010), select RAxML-HPC v.8 on XSEDE (8.2.12) tool, with the default settings but modify some parameters: the GAMMA nucleotide substitution model and 1000 rapid bootstrap replicates.

**Table 1:** Details of sequences used for *Lasiodiplodia crassispora* and *L. pseudotheobromae* phylogenetic analyses.

Species	Voucher/Strain	GenBank accession numbers	
		ITS	*tef*1-α
*Lasiodiplodia acacia*	CBS 136434^T^	MT587421	MT592133
*L. aquilariae*	CGMCC 3.18471^T^	KY783442	KY848600
*L. cinnamomi*	CFCC 51997^T^	MG866028	MH236799
*L. citricola*	CBS 124707^T^	GU945354	GU945340
*L. crassispora*	CBS 118741^T^	DQ103550	DQ103557
*L. crassispora*	CMW 13488	DQ103552	DQ103559
*L. crassispora*	CBS 110492	EF622086	EF622066
*L. crassispora*	**RUFCC2463**	**PQ327547**	**PQ336773**
*L. curvata*	CGMCC 3.18456^T^	KY783437	KY848596
*L. laeliocattleyae*	CBS 130992^T^	JN814397	JN814424
*L. euphorbiaceicola*	CMM 3609^T^	KF234543	KF226689
*L. exigua*	CBS 137785^T^	KJ638317	KJ638336
*L. gilanensis*	CBS 124704^T^	GU945351	GU945342
*L. gonubiensis*	CMW 14077^T^	AY639595	DQ103566
*L. gravistriata*	CMM 4564^T^	KT250949	KT250950
*L. hormozganensis*	CBS 124709^T^	GU945355	GU945343
*L. iraniensis*	CBS 124710^T^	GU945348	GU945336
*L. laosensis*	CGMCC 3.18464^T^	KY783471	KY848609
*L. macrospora*	CMM 3833^T^	KF234557	KF226718
*L. mediterranea*	CBS 137783^T^	KJ638312	KJ638331
*L. parva*	CBS 456.78^T^	EF622083	EF622063
*L. plurivora*	STE-U 5803^T^	EF445362	EF445395
*L. pontae*	CMM 1277^T^	KT151794	KT151791
*L. prunus*	JZB3130029^T^	OR821993	OR831982
*L. pseudotheobromae*	CBS 116459^T^	EF622077	EF622057
*L. pseudotheobromae*	CBS 116460	EF622078	EF622058
*L. pseudotheobromae*	MFLUCC 23-0141	OR438380	N/A
*L. pseudotheobromae*	MFLUCC 20-0137	MT947087	MT951067
*L. pseudotheobromae*	**RUFCC2464**	**PQ327548**	N/A
*L. pyriformis*	CBS 121770^T^	EU101307	EU101352
*L. rubropurpurea*	WAC12535^T^	DQ103553	DQ103571
*L. subglobosa*	CMM 3872^T^	KF234558	KF226721
*L. thailandica*	CBS 138760^T^	KJ193637	KJ193681
*L. theobromae*	CBS 164.96^T^	AY640255	AY640258
*L. vaccinii*	CGMCC 3.19022^T^	MH330318	MH330327
*L. venezuelensis*	CBS 118739^T^	DQ103547	DQ103568
*L. viticola*	CBS 128313^T^	HQ288227	HQ288269
*L. vitis*	CBS 124060^T^	KX464148	MN938928
*Diplodia mutila*	CMW 7060	AY236955	AY236904
*D. seriata*	CBS 112555^T^	AY259094	AY573220

The names, isolate numbers, and corresponding GenBank accession numbers of the taxa used for phylogenetic trees. ^“T”^ denotes ex-type. Newly generated sequences are indicated in bold. “N/A”, no data available in GenBank. The Centraalbureau voor Schimmelcultures (Central Bureau of Fungal Cultures), Fungal and Yeast Collection (CBS), China Forestry Culture Collection Center (CFCC), China General Microbiological Culture Collection (CGMCC), Culture Collection of Phytopathogenic Fungi Prof. Maria Menezes (CMM) at the Federal Rural University of Pernambuco, culture collections (CMW) of the Forestry and Agricultural Biotechnology Institute (FABI) at the University of Pretoria, Jiangsu Key Laboratory of Zoonosis, Yangzhou, Jiangsu, PR China (JZB), Mae Fah Luang University Culture Collection (MFLUCC), Rajarata University Fungal Culture Collection (RUFCC), Culture collection of the Department of Plant Pathology, University of Stellenbosch, South Africa (STE-U), and The Western Australian Plant Pathology Culture Collection (WAC).

Bayesian Inference (BI) analysis was processed via MrBayes v. 3.0b4 (Ronquist and Huelsenbeck, 2003), MrModeltest v. 2.2 was performed to evaluated the model of evolution (Nylander, 2004). MrBayes v.3.0b4 (Huelsenbeck and Ronquist, 2001) was selected, following Markov chain Monte Carlo sampling (MCMC) was used for calculating the posterior probabilities (PP) (Rannala and Yang, 1996; Zhaxybayeva and Gogarten, 2002). Six simultaneous Markov chains were run for 1,000,000 generations, with trees sampled every 100^th^ generation. The preburn was set to 5 and the run was automatically stopped when the mean standard deviation of the split frequency reached below 0.01 (Maharachchikumbura et al., 2015).

ML and BI trees were edited and exported as emf. format by Figtree v. 1.4.0 (https://tree.bio.ed.ac.uk/software/figtree/) (Rambaut, 2006). Maximum likelihood bootstrap values (MLBP) equal or greater than 50% and Bayesian posterior probabilities (BYPP) equal or greater than 0.95 are indicated on the resulting tree topology. The Final improvement of trees’ phylogram was edited by Microsoft Office PowerPoint 2016 (Microsoft Inc., Redmond, WA, USA) and converted to jpg. file using Adobe PhotoShop CC 2018 software (Jiang et al., 2021).

### 2.5 Dye decolourisation on solid media

The solid media were prepared by adding 5 mL of a 0.01% (w/v) concentration of each synthetic dye separately, mixed with 1 L of PDA medium under aseptic conditions (Rani et al., 2014; Ekanayake and Manage, 2022). Tetracycline (50 mg L^-1^) was added to prevent bacterial growth, and sterile media were poured onto Petri plates in volumes measuring about 10 mL each. The media plates were allowed to solidify within a laminar hood chamber under UV (Ultraviolet) exposure, ensuring the enhanced neutralization of any potentially viable bacterial or fungal spores.

Mycelium plugs (diameter ∼5 mm) obtained from the edge of pure cultures of fungal strains grown on PDA medium plates for four days at room temperature (28 °C–30 °C) were transferred to the center of a solid medium plate and inoculated at room temperature for seven to ten days in the dark to prevent polymerization of the dye. Un-inoculated plates containing each dye were maintained under the same conditions as a control. The plates were examined daily for the visual disappearance of colour compared to the control. Fungal strains that exhibited the most substantial colour disappearance during solid media screening were selected for further evaluation in liquid medium screening (Khambhaty et al., 2015). The decolourisation ability of synthetic dyes on solid media was assessed through visual observation every 24 hours until ten days after fungal inoculation. The Munsell colour notation system, including hue, chroma, and value, was used to visually detect the disappearance of synthetic dye discolouration, aiding in the identification of colour changes in synthetic dyes in solid media (Yi and Cho, 2008; Raluca, 2016). The visual disappearance of colour on both the up and down sides of the plate indicated the decolourisation ability of the fungal strains.

### 2.6 Dye decolourisation in liquid media

The decolourisation of dyes in the liquid phase was evaluated using 1 L of PDB medium containing tetracycline (50 mg L^-1^) and 5 mL of a 0.01% (w/v) concentration of each synthetic dye separately. Falcon tubes containing 30 mL of liquid medium with each dye were inoculated with five agar plugs of mycelia (diameter ∼5 mm) obtained from the edge of actively growing colonies on pure culture plates containing the selected fungi. The liquid cultures were incubated at 30 °C for four weeks in a shaking incubator (180 rpm) (Jayasinghe et al., 2008; Purnomo et al., 2019). Un-inoculated falcon tubes, each containing liquid medium and dye, were employed as controls. Each set undergoes a liquid media dye decolourisation assay conducted in triplicate.

After incubation, falcon tubes were centrifuged at 4000 rpm for 10 min. to obtain biomass and supernatant, which were measured using a UV-VIS spectrometer (Thermo Scientific Multiskan GO with cuvette 100–240 V, SN 1510-049220, Ratastie 2, Fl 01620 Vantaa, Finland). Changes in absorbance were recorded at the maximum wavelength ranges of 585 nm for CV, 500 nm for CR, 520 nm for Safranin, and 625 nm for MG, respectively. The absorbance values were used to calculate the percentage of decolourisation (%DP) based on the equation (Purnomo et al., 2019; Ekanayake et al., 2022).


$${\text{DP (\%) = [(Abs}}_{0}{\text{-Abs}}_{\text{t}}{\text{)/Abs}}_{0}]\times 100$$

$${\text{Abs}}_{0}\;-\text{ control absrobance, and }{\text{Abs}}_{\text{t}}-\text{treatment absorbance}$$

### 2.7 Data analysis

Experimental data from three replicates in the liquid media assay were statically analysed using a one-way ANOVA with Duncan’s t-test, applying SPSS software (version 25.0), with a significance level set at *p *< 0.05. Principal Component Analysis (PCA) was performed using R software (https://www.r-project.org/) to interpret the data.

### 2.8 Development of the prototypes for synthetic dye decolourisation

To address the challenge of synthetic dye decolourisation, we designed a scalable prototype leveraging the mycoremediation potential of *Lasiodiplodia pseudotheobromae* for CV decolourisation. The development process focused on optimizing critical growth conditions for the fungal species, including shaking speed, temperature, pH, nutrient composition, and surface sterilisation techniques, to maximize decolourisation efficiency. The prototype was developed in two stages, with the first-stage design serving as the basis for improvements incorporated into the second-stage system.

#### 2.8.1 First stage of prototype development

Based on the fundamental concept of prototype development (described in Section 4.1), the first-stage prototype was designed using SolidWorks software (https://www.solidworks.com; accessed on 10 April 2024). The system comprised four interconnected PP5 plastic containers, ball valves, pneumatic plastic pipes, and a wooden frame. The design utilised potential energy to enable gravity-driven water flow (Jones, 2011; Babonneau et al., 2019; Zhang et al., 2019), eliminating the need for additional energy consumption from pumps. The containers were arranged in a descending, stair-like configuration within the wooden frame to ensure smooth and continuous water transfer between stages.

Potato Dextrose Broth was used as the primary medium for fungal growth, supplemented with tetracycline (50 mg L^−1^) to prevent bacterial contamination. The PDB medium was sterilised prior to dye addition. To prepare the dye mixture, 5 mL of a 0.01% (w/v) CV solution was membrane-filtered and added to 1 L of sterilised PDB. For optimization of fungal growth and CV decolourisation, shaking speeds were tested from high (180 rpm) to low (90 rpm), incubation temperature was evaluated between 28 °C and 32 °C, pH was monitored in each container, and the nutrient composition of PDB (20% potato, 2% dextrose) was standardised. The total decolourisation process was monitored over 21 days. For further details on the first-stage prototype development, see Section 4.2.

#### 2.8.2 Second stage of prototype development

The second-stage prototype was developed to address the limitations observed in the initial design, including low water storage capacity, limited durability, and impractical agitation for larger-scale applications. Using SolidWorks 2022 (accessed on January 5, 2025), the system layout was optimised to enhance scalability, durability, and operational efficiency. The prototype consists of five capsule chambers, two serving as temporary wastewater storage tanks, one for the collection of treated wastewater, and three directly integrated into the automated system, which provides continuous shaking at 90 rpm with an incubation temperature of 30 °C. The system incorporates borosilicate glass treatment chambers, copper valves, and polypropylene tubing to ensure chemical and thermal resistance, allowing for heat sterilisation by autoclaving. An electronically controlled shaking mechanism using NEMA stepper motors delivers continuous, bi-directional agitation, with independent control of shaking speed and direction. The primary medium (PDB), its nutrient composition, the tested dye (CV) and its concentration, as well as the antibacterial treatment, were the same as those used in the first-stage prototype (see Section 2.8.1).

**Figure 2:**
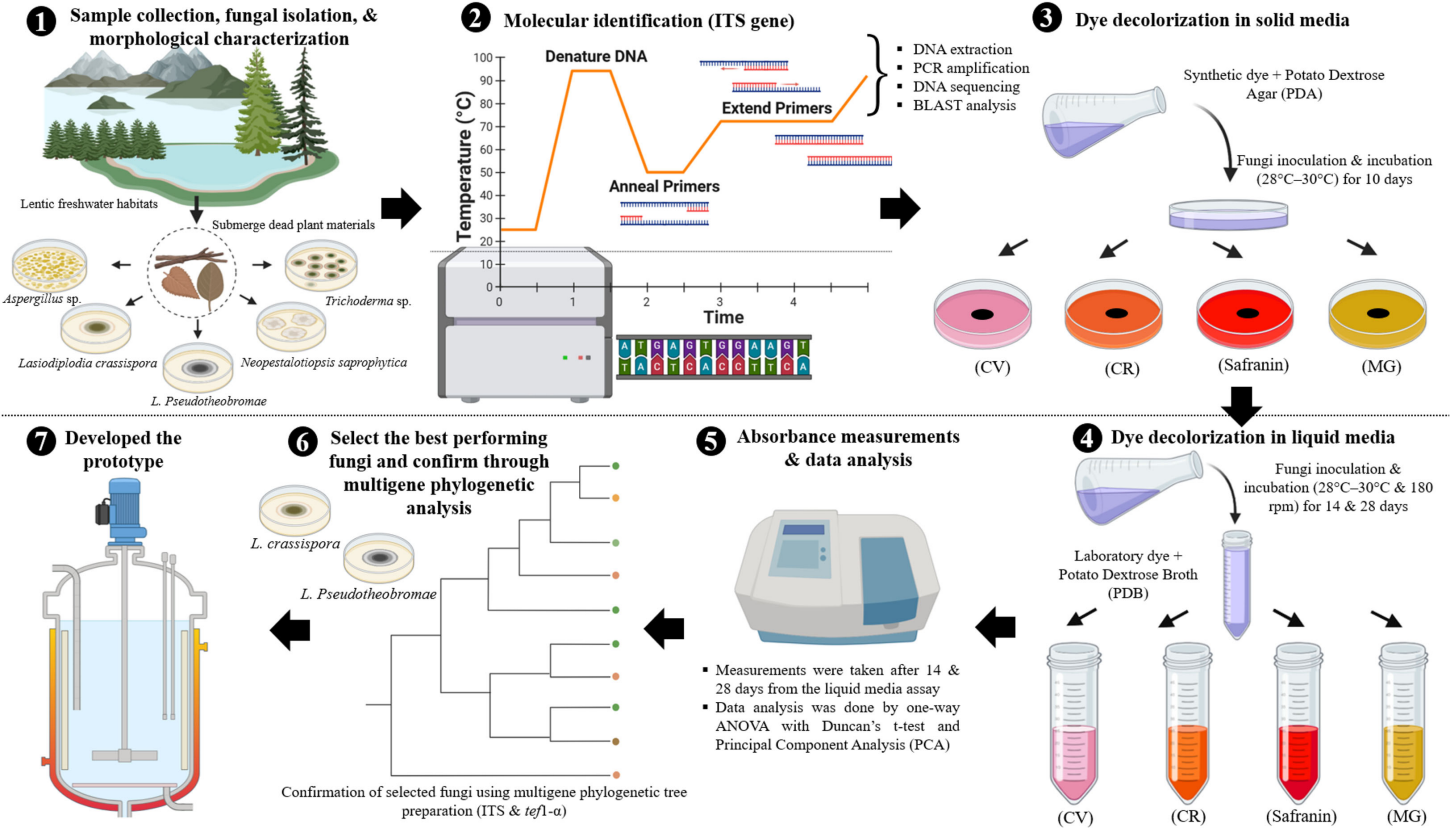
A summarised graphical representation of the experimental methodology for synthetic dye decolourisation by fungi. (The figure was created using BioRender.com).

Operationally, the temporary wastewater chamber was first filled with dye-containing PDB. The dye-containing PDB was then transferred to the second chamber, which was connected to the automated system. In this chamber, 30 g of pre-cultured mycelium (∼5 mm diameter) was added, and the contents were incubated for five days under shaking at 90 rpm and 30 °C to facilitate decolourisation. Following this period, the water was sequentially circulated to the subsequent capsules. The complete decolourisation process required 15 days, with five days of treatment in each of the three active capsules. The third and fourth capsule chambers were maintained under the same conditions as described for the second chamber. Sequential circulation enabled the collection of clear, decolourised water from the final chamber. For further details on the second-stage prototype development, see Section 4.3.

(Note: **Figure 2** presents a summarised graphical representation of the experimental methodology for synthetic dye decolourisation by fungi. The figure was created using BioRender.com).

## 3 Results

### 3.1 Screening of the synthetic dye decolourisation ability of fungi on solid media

Five fungal strains isolated from freshwater habitats in Sri Lanka were identified and confirmed as *Aspergillus* sp., *Lasiodiplodia crassispora*, *L. pseudotheobromae*, *Neopestalotiopsis saprophytica*, and *Trichoderma* sp. using morphological and molecular characteristics. These strains were selected for the dye decolourisation experiment based on their rapid growth rate in the PDA medium within four days.

#### 3.1.1 Crystal Violet decolourisation on solid media

All five selected fungal species (*Lasiodiplodia crassispora*, *L. pseudotheobromae*, *Aspergillus* sp., *Neopestalotiopsis saprophytica*, and *Trichoderma* sp.) decolourised CV. Among the tested fungal strains, *L. pseudotheobromae* demonstrated the highest decolourisation efficiency for CV dye, altering its colour to closely resemble that of the PDA medium.

#### 3.1.2 Congo Red decolourisation on solid media

Congo Red was effectively decolourised by *Lasiodiplodia crassispora*, *L. pseudotheobromae*, and *Aspergillus* sp. while *Neopestalotiopsis saprophytica* and *Trichoderma* sp. did not decolourise CR dye.

#### 3.1.3 Malachite Green decolourisation on solid media

Malachite Green was efficiently decolourised by *Lasiodiplodia crassispora* and *L. pseudotheobromae*, whereas other fungal strains did not exhibit decolourisation ability on a solid medium. MG synthetic dye acted as a growth inhibitor for *Aspergillus* sp., *Neopestalotiopsis saprophytica*, and *Trichoderma* sp. and hindered proper growth on solid media when compared to other dyes.

#### 3.1.4 Safranin decolourisation on solid media

Safranin was effectively decolourised by *Lasiodiplodia crassispora*, while *L. pseudotheobromae*, *Neopestalotiopsis saprophytica* and *Aspergillus* sp. visually exhibited the absorption of safranin into their mycelia, resulting in a reddish-pink colour. However, *Trichoderma* sp. grew well on solid media containing safranin, with no significant differences observed in the dye remediation on solid media.

**Figure 3:**
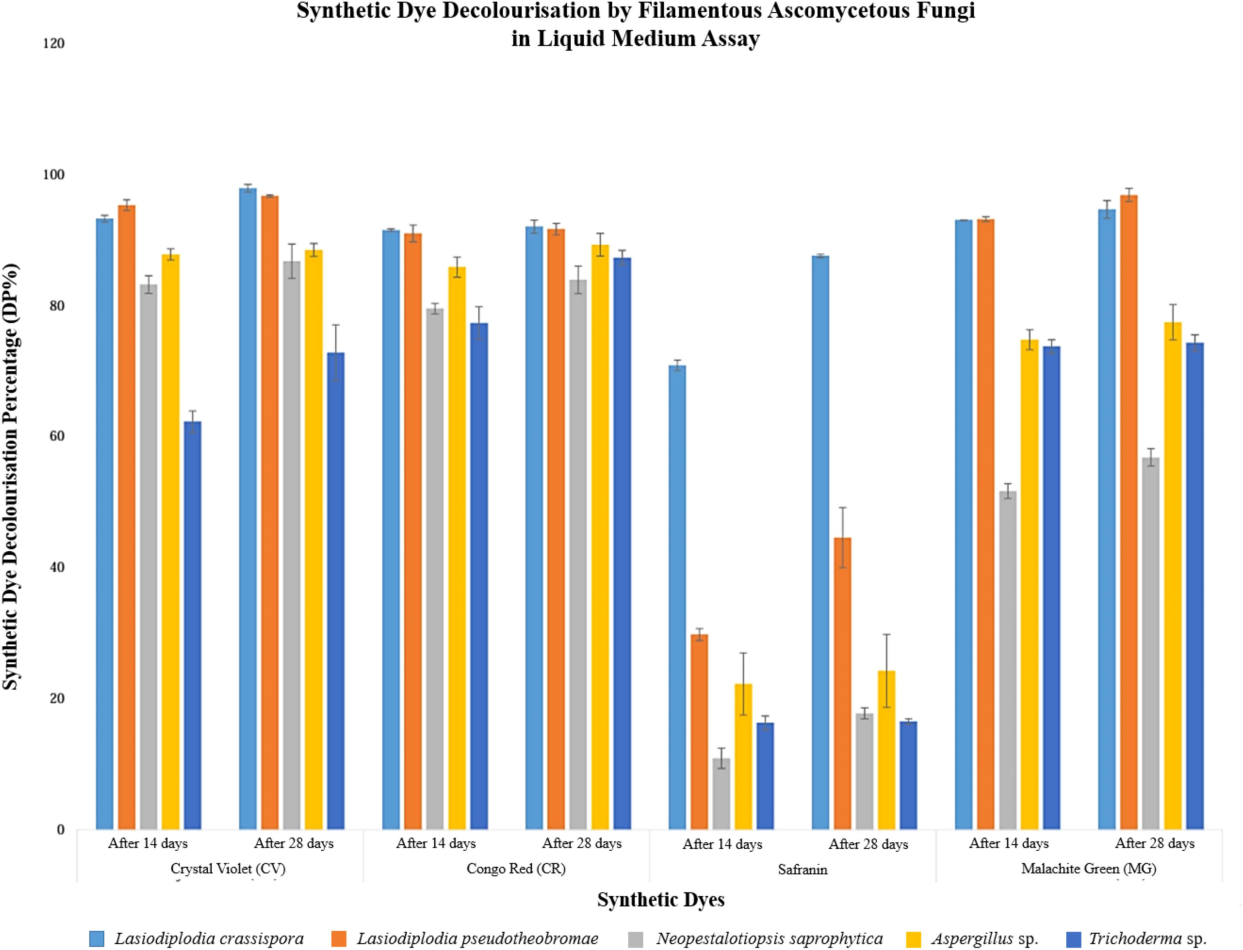
Graphical representation of synthetic dye decolourisation by filamentous ascomycetous fungi in liquid medium assay (14 and 28 days after fungal inoculation).

Overall, the identification results indicated that *L. crassispora* significantly decolourised all selected dyes, and all isolated fungal strains effectively decolourised CV on a solid medium. The decolourisation ability of *L. crassispora*, *L. pseudotheobromae*, and *Aspergillus* sp. on solid media was also confirmed through the liquid media decolourisation assay (with > 85% DP), as shown in **Figure 3**.

### 3.2 Screening of the synthetic dye decolourisation ability on liquid media based on visual observation

Visual observation facilitated the comparison of synthetic dye colour variations between control and treated samples, revealing significant changes in colour after 14 and 28 days of decolourisation.

#### 3.2.1 Crystal Violet decolourisation on liquid media, as determined by visual observation

The samples treated with *Lasiodiplodia crassispora*, *L. pseudotheobromae*, *Aspergillus* sp., *Neopestalotiopsis saprophytica*, and *Trichoderma* sp. displayed a noticeable decrease in colour compared to the control samples. Among these observations, all the tested fungal strains displayed the most significant colour variation with CV compared to other treated samples (**Supplementary Figure 1**).

#### 3.2.2 Congo Red decolourisation on liquid media, as determined by visual observation

In the liquid media, a significant reduction in colour was observed for samples treated with CR across all isolated fungal strains. However, *Lasiodiplodia crassispora*, *L. pseudotheobromae*, and *Aspergillus* sp. showed significant colour variations compared to other treated samples in the context of CR (**Supplementary Figure 2**).

#### 3.2.3 Safranin decolourisation on liquid media, as determined by visual observation

Safranin decolourisation was observed in *Lasiodiplodia crassispora*, *L. pseudotheobromae*, *Neopestalotiopsis saprophytica*, and *Aspergillus* sp. in comparison with the control and other treated samples (**Supplementary Figure 3**).

#### 3.2.4 Malachite Green decolourisation on liquid media, as determined by visual observation

Visual observation was used to compare the decolourisation of MG in samples of *Lasiodiplodia crassispora*, *L. pseudotheobromae*, and *Aspergillus* sp. and to compare the colour variation of other treated samples with MG (**Supplementary figure 4**).

### 3.3 Screening of the synthetic dye decolourisation ability on liquid media based on UV-VIS spectrophotometry

#### 3.3.1 Fungal decolourisation efficiency based on one-way ANOVA with Duncan’s t-test

In liquid medium, the highest decolourisation percentage was reported for CV and MG (95.23% and 93.12%, respectively) in *Lasiodiplodia pseudotheobromae* after 14 days of incubation. *Lasiodiplodia crassispora* demonstrated the highest decolourisation percentage for CR and Safranin, with 91.45% and 70.83% dye decolourisation, respectively after 14 days of incubation.

After 14 days of fungal inoculation into the liquid media, *Aspergillus* sp., *Lasiodiplodia crassispora*, *L. pseudotheobromae*, and *Neopestalotiopsis saprophytica* demonstrated robust decolourisation performance, each surpassing 85% in CV. Similarly, *Aspergillus* sp., *L. crassispora*, and *L. pseudotheobromae* demonstrated superior decolourisation, surpassing 85% in CR.

Furthermore, *Lasiodiplodia crassispora* and *L. pseudotheobromae* showcased remarkable efficacy, reporting over 85% decolourisation in MG. When comparing the results after 28 days with those after 14 days, it was observed that all synthetic dyes were effectively decolourised, surpassing the reported data from the initial 14 days of fungal inoculation into liquid media. Considering the overall results, all fungal strains successfully achieved more than 60% decolourisation of CV, CR, and MG. However, Safranin decolourisation was inadequately recorded in isolated fungal strains, except for *L. crassispora*. **Figure 3** provides a graphical representation of synthetic dye decolourisation by filamentous ascomycetous fungi in a liquid medium assay, recorded at 14 and 28 days after fungal inoculation. Error bars indicate the standard deviation.

#### 3.3.2 Fungal decolourisation efficiency based on Principal Component Analysis

The position of each dye indicates the efficacy of decolourisation by each fungus, represented by two sets of points for each fungal species, one at 14 days and another at 28 days, demonstrating the temporal changes in decolourisation. PC1 accounts for 77.42% of the variance, capturing the majority of differences in dye decolourisation, while PC2 explains 12.56%. In addition, PC1 and PC2 represent linear combinations of dyes, where MG and Safranin have a strong influence, whereas CV and CR have a negative influence.

*Lasiodiplodia crassispora* and *L. pseudotheobromae* are positioned on the positive side of PC1, indicating strong decolourisation ability, especially for CV, MG, and Safranin. Their shift from 14-day to 28-day points suggests improved decolourisation over time, with the 28-day points moving farther from the origin. *Aspergillus* sp. is closer to the origin but still positive on PC1 and shows moderate decolourisation potential. Although their decolourisation ability improves over time, it is less dramatic compared to *Lasiodiplodia crassispora* and *L. pseudotheobromae.*

**Figure 4:**
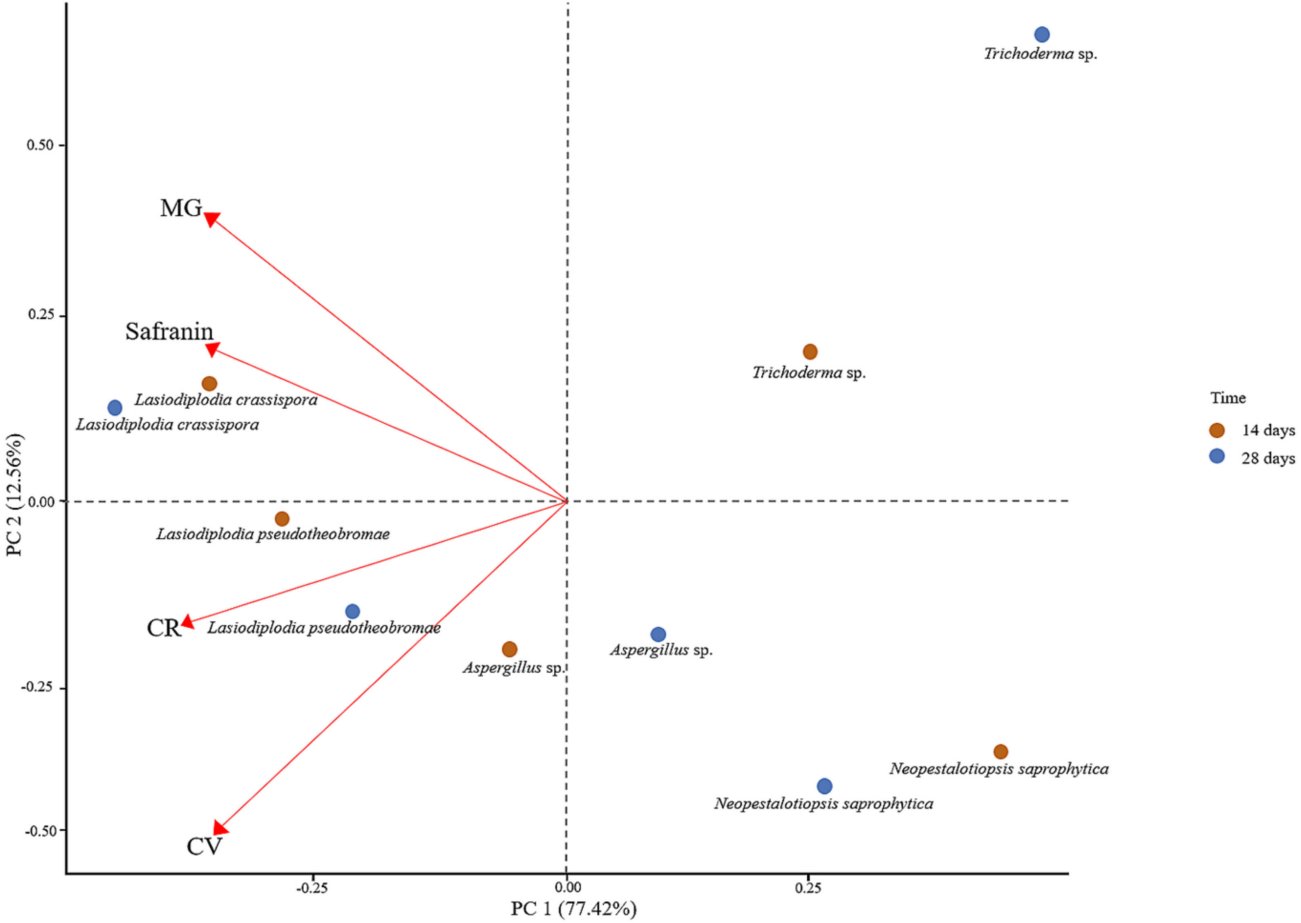
Principal component analysis (PCA) for assessing the decolourisation ability of fungi on laboratory dyes.

*Neopestalotiopsis saprophytica* also appears on the positive side of PC1, indicating moderate to low decolourisation capacity, without a strong preference for specific dyes. *Trichoderma* sp., while showing good overall decolourisation ability, is positioned between *Aspergillus* sp. and *Lasiodiplodia* spp. (*L. crassispora* and *L. pseudotheobromae*), indicating it is not as effective as *L. crassispora* and *L. pseudotheobromae*. Based on their overall performance, *L. crassispora* and *L. pseudotheobromae* are the most effective decolourizers across the tested dyes, showing marked improvement over time, especially with CV, MG, and Safranin. Their broad decolourisation capacity and consistent enhancement with prolonged exposure make them the top performers (**Figure 4**).

Overall, the plot has emphasised the importance of understanding the impact of specific dyes on the decolourisation ability of various fungal species over time.

### 3.4 Phylogenetic analyses for precise identification of the effective strains in dye decolourisation: *Lasiodiplodia crassispora* and *L. pseudotheobromae*

**Figure 5:**
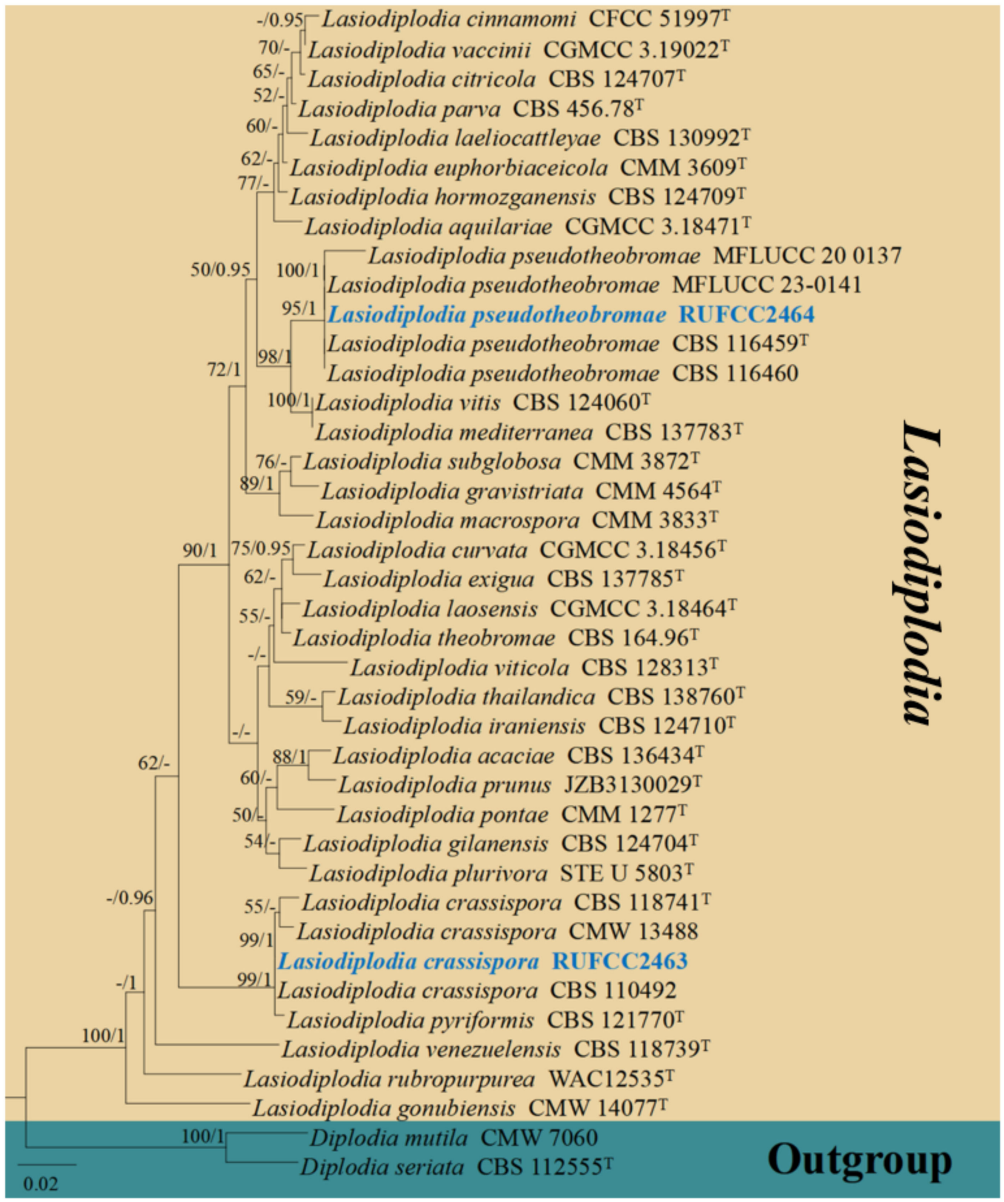
Phylogenetic tree from the best scoring of the RAxML analysis based on combined (ITS, and *tef*1-α is rooted to *Diplodia mutila* (CMW 7060) and *D. seriata* (CBS 112555). Bootstrap values for maximum likelihood and Bayesian posterior probabilities (BYPP) equal to or greater than 50% and 0.95, are given at the respective branches. Hyphen (-) means a value lower than 50% (MLBP) or 0.95 (BYPP). Ex-types are marked in ^“T”^. New isolates are labelled in bold and blue.

The concatenated dataset (ITS and *tef*1-α regions) contained 40 strains in the sequence analysis, which comprise 750 characters with gaps. Single gene analysis was carried out and compared with each species, to compare the topology of the tree and clade stability. *Diplodia mutila* (CMW 7060) and *D. seriata* (CBS 112555, ex-type) are set as the outgroup taxa. The best-scoring RAxML tree with a final likelihood value of -3104.886120 is presented. The matrix had 217 distinct alignment patterns, with 4.91% of undetermined characters or gaps. Estimated base frequencies were as follows; A = 0.215130, C = 0.285729, G = 0.257826, T = 0.241315; substitution rates AC = 0.865801, AG = 4.151570, AT = 1.525331, CG = 1.008008, CT = 5.192424, GT = 1.000000; gamma distribution shape parameter alpha = 0.159782 (**Figure 5**). GTR+I+G model was selected as the best model based on MrModeltest and was used for the Bayesian analysis. Overall, tree topologies based on ML and BI analyses were similar and not significantly different. In the phylogenetic analysis (**Figure 5**), our new collections RUFCC2463 and RUFCC2464 grouped with *Lasiodiplodia crassispora* (CBS 118741 (ex-type), CMW 13488 and CBS 110492) and *L. pseudotheobromae* (CBS 116459 (ex-type), CBS 116460, MFLUCC 23–0141 and MFLUCC 20-0137) with high statistical support (99% MLBP, 1.00 BYPP) and (95% MLBP, 1.00 BYPP), separately.

Based on the current *in-vitro* experiments, the strain numbers RUFCC2463 and RUFCC2464 have been revealed as effective in the decolourisation process. Mega blast results in the GenBank regarded these strains as belonging to *Lasiodiplodia crassispora* (strain numbers: RUFCC2463) and *L. pseudotheobromae* (strain numbers: RUFCC2464). We carried out multi-gene analyses based on ITS (ITS4/ITS5), and *tef*1-α (EF-725F/EF-986R) gene regions for further confirmation (**Figure 5**). Despite numerous adjustments to the PCR time range and annealing temperatures, *L. pseudotheobromae* failed to amplify using *tef*1-α. Consequently, the phylogenetic analysis for *L. pseudotheobromae* was based solely on the ITS locus.

### 3.5 Selection of the best-performing fungi for dye decolourisation in the prototype

Based on liquid and solid media assays, *Lasiodiplodia pseudotheobromae* was identified as the most effective of the five tested fungal strains for CV decolourisation and was subsequently applied in the prototype. Apparently, it is essential to identify the species precisely before its applications; thus, here we provide the identification procedure and taxonomy (microscopy and molecular phylogenetics) of the newly isolated *Lasiodiplodia pseudotheobromae*.

### 3.6 Taxonomy and classification of *Lasiodiplodia pseudotheobromae*

*Ascomycota* Caval.-Sm

*Dothideomycetes* O.E. Erikss. & Winka

*Botryosphaeriales* C.L. Schoch, Crous & Shoemaker

*Botryosphaeriaceae* Theiss. & Syd.

*Lasiodiplodia* Ellis & Everh.

Notes: The genus *Lasiodiplodia* showed 94 records in Index Fungorum 2025 (https://www.indexfungorum.org/names/Names.asp; accessed on 12^th^ June 2025). Species belonging to the genus *Lasiodiplodia* are widely distributed in tropical and subtropical regions and are increasingly documented in temperate areas (Jayawardena et al., 2019; Rathnayaka et al., 2023). Since 2004, over 70 *Lasiodiplodia* species have been identified and characterised based on morphology and molecular data (Ko et al., 2023). The genus has been extensively studied for its pathogenic impact on numerous significant agricultural plants, with thousands of host plants recorded (Gunamalai et al., 2023; Ko et al., 2023).

*Lasiodiplodia pseudotheobromae* A.J.L. Phillips, A. Alves & Crous, Fungal Diversity 28: 8 (2008)

Index Fungorum Registration Identifier: 510941

Description: Saprobic on a submerged woody stem. Sexual morph: Undetermined. Asexual morph: *Conidiomata* 190–300 µm high × 120–230 µm diam. (*x̅* = 240 × 170 µm, n = 10), pycnidial, semi-immersed, unilocular, solitary, scattered, globose or subglobose, dark brown. *Conidiomata wall* thick, outer layers dark brown to black, inner layers thin-walled, pale brown to hyaline, comprising 2–3 layers of dark brown cells of textura angularis. *Conidiogenous cells* 20–35 × 5.5–10 µm (*x̅* = 25 × 7 µm, n = 10), holoblastic, hyaline, thin-walled, smooth, cylindrical. *Conidia* 28–50 µm × 11.5–14 μm (*x̅* = 38 × 12.5 µm, n = 30), initially hyaline and aseptate when immature, becoming medianly one-septate, dark brown, thick-walled, ellipsoid to obovoid, base truncate or rounded, with longitudinal striations from apex to base.

Culture characteristics: Initially, the colonies on the plate appeared white with sparse, fluffy aerial mycelia on the surface. After one week, they developed a pale grey colour with dark pigmentation, eventually turning greyish-black. The reverse side of the plate also displayed a greyish-black colouration after a seven-day incubation period at 28–30 °C.

Material examined: SRI LANKA, North Central Province, Anuradhapura District, Mihintale, Mihintale tank (8.38683° N, 80.38683° E, 117 m), on Submerged woody stem, 10^th^ January 2023, Madhara K. Wimalasena, living culture (RUFCC2464).

**Figure 6:**
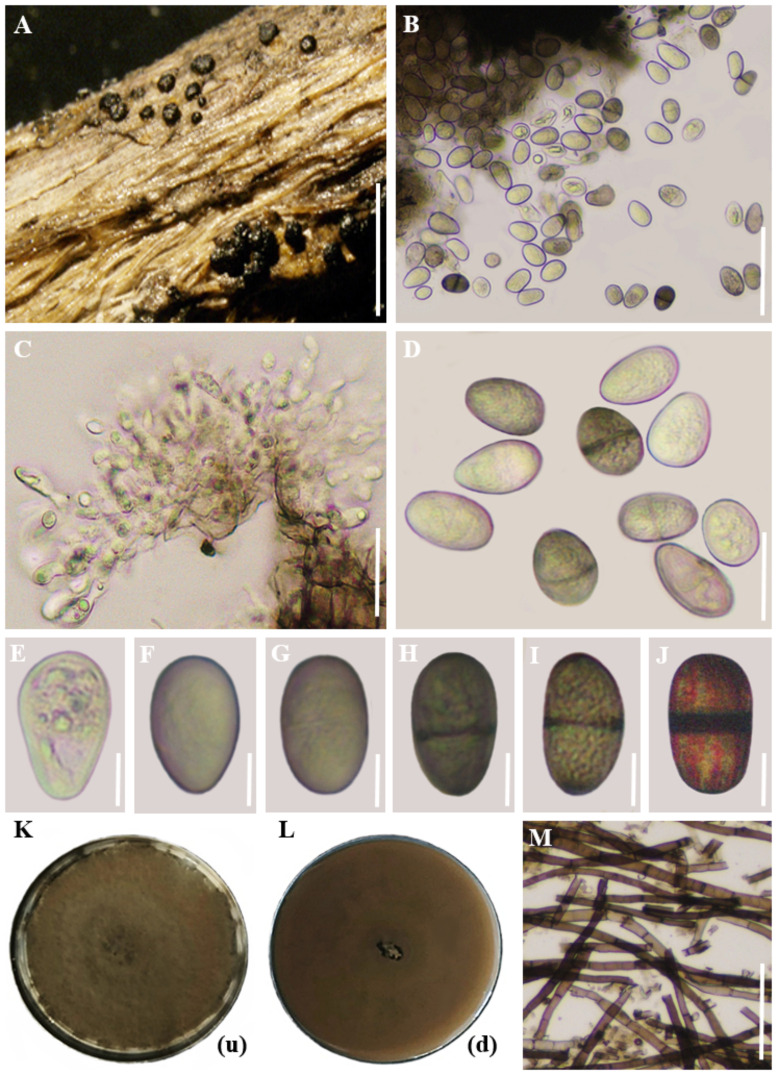
Morphological and colony characteristics of *Lasiodiplodia pseudotheobromae.* **(A)** Appearance of conidiomata on twig. **(B)** Vertical sections through conidiomata. **(C)** Conidiogenous cells. **(D)** Conidia. **(E-J)** Maturing stages of conidia. (K, L) Colony growth on PDA medium after 10 days (up and downsides). (M) Mycelium. Scale bars: **(A)** = 500 µm, **(B–D)**, **(M)** = 20 µm, **(E–J)** = 10 µm. (Note: In images **(K)** and **(L)**, the letters within brackets denote the orientation of the plate, where (u) represents the upper side and (d) indicates the lower side).

**Figure 6** represents the morphological and colony characteristics of *Lasiodiplodia pseudotheobromae.*

## 4 Development of a prototype for synthetic dye decolourisation

### 4.1 Basic concept for the development of a prototype

**Figure 7:**
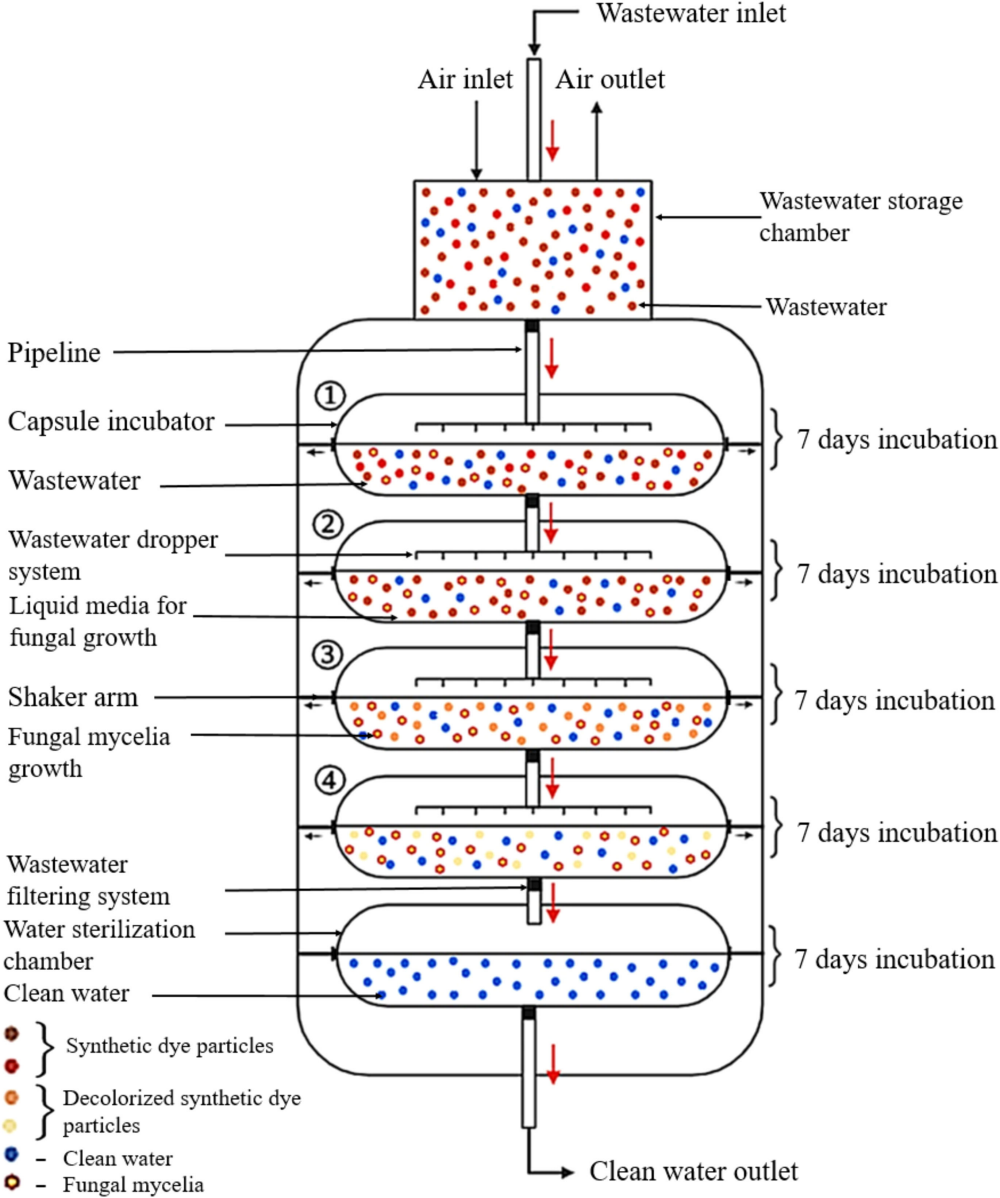
The microencapsulation system for synthetic dye decolourisation in wastewater treatment plants. (The figure was created using BioRender.com).

Mycoremediation to remove synthetic dyes should be extended to outdoor settings, particularly in industrial wastewater plants that use synthetic dyes. The treated wastewater must be released into the environment without causing any adverse effects from using fungal strains. Therefore, it is important to design the mycoremediation system for future implementations. An exciting prospect is the use of capsule mycoremediation in the process of removing synthetic dyes (**Figure 7**).

This innovative system is composed of a temporary wastewater storage chamber, capsule incubators, and a water sterilisation chamber. The wastewater, which contains synthetic dyes, flows from the temporary storage tank to the capsule incubator through a filtering system that removes debris before releasing the water into the capsule chamber. The capsule incubator receives wastewater through a dropper system from the temporary storage chamber, which allows the wastewater to be released into the liquid media without causing damage to the fungal mycelia growth. The system comprises four capsule chambers, each containing a liquid medium suitable for specific fungal growth. The incubation temperature within the liquid media are automatically adjusted according to standard parameters specific to the growth requirements of each fungal strain. To ensure proper fungal mycelia growth, fungal inoculation is carried out seven days prior to operating the capsule mycoremediation. Each capsule chamber retains the wastewater for seven days with periodic shaking. After this period, the treated wastewater is automatically transferred to the second capsule chamber through the filtering system. The second capsule chamber holds the water for an additional seven days for synthetic dye remediation and then automatically advances to the third chamber. The third and fourth chambers further decolourize the wastewater using the previously described process. In the water sterilisation chamber, the treated wastewater is held for two days while undergoing sterilisation through either heat sterilisation or chlorinated water. This step directly contributes to eliminating any harmful effects that occur in the environment resulting from using fungi in the remediation process.

### 4.2 First stage of prototype development

Based on assays conducted on both solid and liquid media, *Lasiodiplodia pseudotheobromae* was selected for the prototype application due to its superior ability to decolourise CV compared to the other fungal isolates tested. Crystal Violet was chosen for the prototype because it exhibited the most rapid and consistent decolourisation among the dyes evaluated.

During the development of the first-stage prototype, one of the primary challenges was providing adequate agitation for fungal growth. This was particularly important because PDB, the main medium used in the prototype, had a tendency to clot if proper shaking was not maintained. Additionally, since the PDB contained CV, thorough mixing was critical to ensure both effective fungal growth and dye exposure. As the initial design lacked a built-in mechanical shaker, the prototype was placed on an orbital shaker (Stuart SI600 Incubator, manufactured by Cole-Parmer Ltd., Beacon Rd, Stone ST15 0SA, United Kingdom), which enabled controlled shaking speed and temperature conditions to optimize fungal development and dye degradation.

Shaking speeds were tested from 180 rpm to 90 rpm, with 90 rpm found to provide the most favorable conditions for mycelial growth. Temperature trials conducted between 28 °C and 32 °C revealed that 30 °C supported the most vigorous fungal development, whereas growth was slower at 28 °C and declined rapidly at 32 °C. Under these optimised conditions, the average pH of the PDB medium was 6.5, supporting efficient dye decolourisation.

The prototype containers were initially designed to retain dye-contaminated water for seven days, with the total experimental period planned for 21 days (seven days per container across four containers). However, visual observations demonstrated that CV was completely decolourised within just four days in the first container. Consequently, the total retention period for the prototype was revised to 16 days instead of 21.

The containers and other components of the prototype were tested to ensure they could be sterilised by autoclaving. All containers and relevant parts were sterilised by autoclaving before use. The PDB medium was also sterilised prior to the addition of dye. To prepare the dye mixture, 5 mL of a 0.01% (w/v) CV solution was added to 1 L of sterilised PDB. Importantly, the CV introduced PDB mixture was not autoclaved, as this could reduce dye intensity. To minimize contamination during CV solution preparation, autoclaved distilled water was used, and the dye solution was sterilised by membrane filtration to remove microbial contaminants.

The first-stage prototype system comprised four plastic containers (each 16 cm × 10 cm × 5 cm) connected via ball valves and pneumatic pipes. These containers were mounted on a treated hardwood frame in a stepwise arrangement, allowing passive water flow between containers using gravity. This mechanism, as described by Jones (2011), reduces energy consumption and enhances the eco-friendliness and sustainability of the prototype for wastewater treatment.

**Figure 8:**
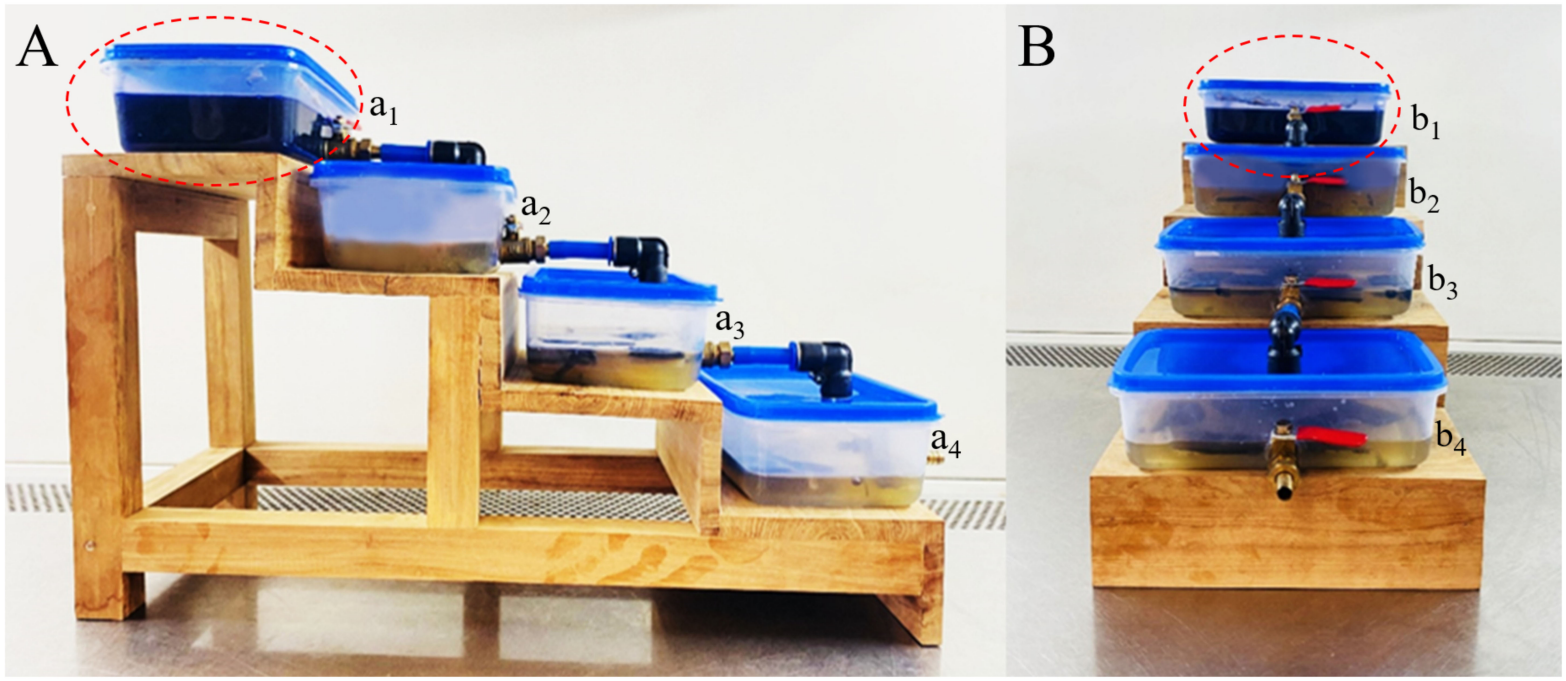
First stage prototype system for assessing CV decolourisation by *L. pseudotheobromae*. **(A)** Side view of the prototype. **(B)** Front view of the prototype. (a_1_, b_1_) Tank 01 containing fungal mycelium actively engaged in CV remediation. (a_2_, b_2_) Tank 02 holding previously treated CV-contaminated water, exhibiting diminished blue colouration after 7 days of incubation. (a_3_, b_3_) Tank 03 containing further decolourised CV-contaminated water following 14 days of incubation. (a_4_, b_4_) Tank 04 displaying significant decolourisation of CV-contaminated water after 21 days of incubation. The red circle highlights CV-containing water, characterised by its blue colouration.

Each container operated sequentially in the decolourisation process. Initially, dye-contaminated water was added to the first container, and 10 g of pre-cultured mycelial plugs (~5 mm diameter) of *L. pseudotheobromae* were introduced. The entire prototype was maintained on an orbital shaker at 90 rpm with 30 °C to ensure optimal fungal activity. The first container was treated for four days, after which the water flowed into the second container via the gravity-driven mechanism. Fresh mycelium plugs were added at each stage, continuing until the dye-degraded water exited through the final ball valve. This stepwise design ensures thorough treatment of CV contaminated water and effective fungal activity at each stage (**Figure 8**). Before releasing the treated water from the fourth container into the environment, it was autoclaved to prevent any potential adverse effects of *L. pseudotheobromae* on the ecosystem.

### 4.3 Second stage of prototype development and redesign

The first stage prototype demonstrated the potential for fungal dye decolourisation wherein several limitations became apparent. The water storage capacity of the system was low, preventing continuous filtration and durability. The use of an orbital shaker, while effective in small-scale testing, was impractical for larger-scale applications, and the overall efficiency of the process was found to be limited.

**Figure 9:**
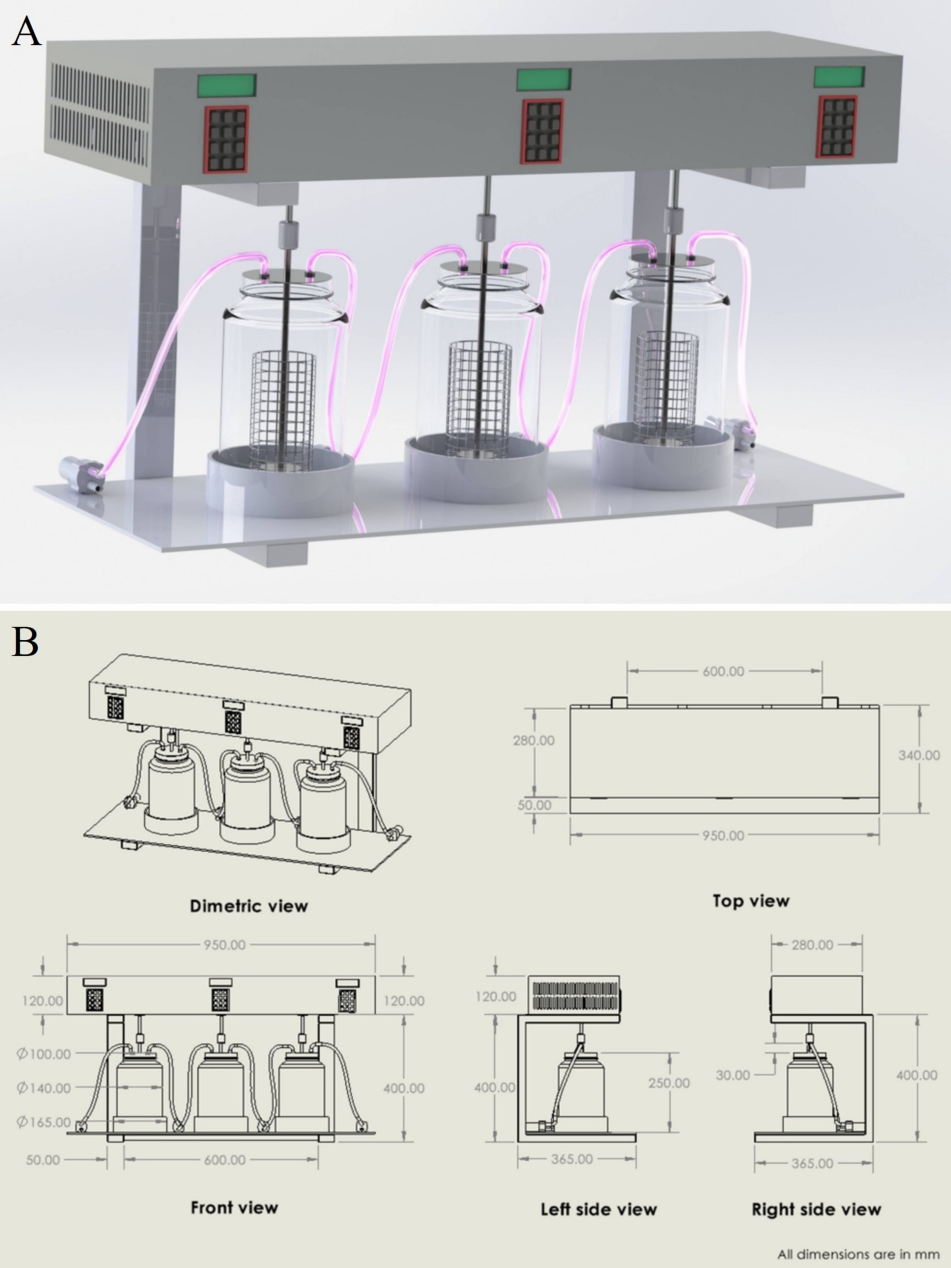
**(A)** 3D design of the second-stage fungal dye decolourisation prototype developed using SolidWorks 2022. The system comprises three borosilicate glass chambers connected via polypropylene tubing, integrated with an electronically controlled shaking mechanism for optimised agitation. **(B)** 2D technical drawing of the second-stage prototype showing dimetric, top, front, and side views with all relevant dimensions in millimetres. The schematic was generated using SolidWorks 2022 to support fabrication and design verification.

The second-stage prototype was designed using SolidWorks 2022 software (accessed on January 5, 2025) to identify design requirements and optimize the system layout, as shown in **Figure 9**.

**Figure 10:**
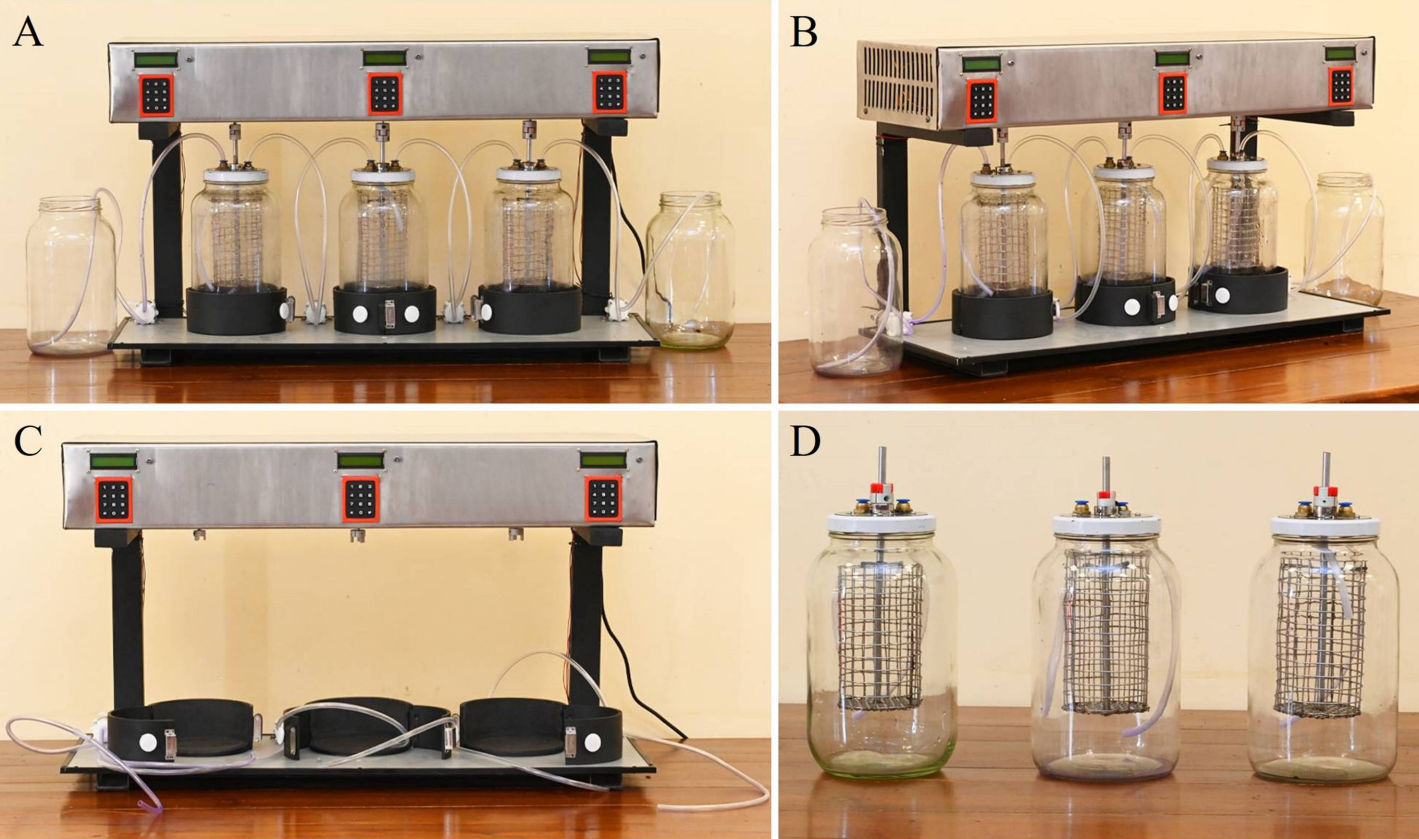
Second stage prototype of the second-stage mycoremediation system for synthetic dye decolourisation. **(A)** Front view of the fully assembled prototype, showing the three treatment capsules connected to the pumping system and control unit. **(B)** Side view illustrating the layout of borosilicate glass chambers, electronic control units, and connecting tubes. **(C)** Stand and electronic mechanism of the prototype without the borosilicate glass chambers, highlighting the modular design. **(D)** Individual borosilicate glass chambers equipped with stainless steel mesh baskets and top-mounted tubing inlets, shown separately from the main stand and electronics.

To overcome these challenges, a second-stage prototype was developed (**Figure 10**), incorporating several key improvements. The new design replaced the plastic containers with larger borosilicate glass bottles (3-L capacity) serving as treatment chambers, copper valves and polypropylene lines, which are commonly used in laboratory-scale fluid storage, transport and wastewater treatment setups due to their cost-effectiveness, chemical resistance and thermal resistance (Kutz, 2002). An electronically controlled shaking mechanism was introduced for a continuous agitation mechanism. This system uses NEMA stepper motors programmed to control both speed and bi-directional rotation (clockwise and counterclockwise), preventing the mycelium from blending while ensuring optimal agitation for dye degradation. The prototype was designed with an integrated water pumping system to facilitate continuous circulation. The system comprises three treatment capsule chambers connected via pumps: the first pump transfers dye-containing water from the storage tank to the initial capsule chamber, while subsequent pumps ensure regulated transfer among the first, second, and third capsules. The second-stage design further incorporates independent control of agitation speed and direction for each tank, enabling a more efficient and continuous decolourisation process.

For operational implementation, the temporary wastewater chamber was initially filled with dye-containing PDB, to which 30 g of pre-cultured mycelial plugs (~5 mm diameter) of *Lasiodiplodia pseudotheobromae* were added. The dye mixture was prepared by adding 15 mL of a 0.01% (w/v) CV solution, sterilised by microbial membrane filtration, to 3 L of sterilised PDB and thoroughly mixing. This medium was subsequently transferred to the second chamber, integrated into the automated system, and incubated for five days under shaking at 90 rpm and 30 °C to promote decolourisation. Following this period, the water was sequentially circulated through the remaining capsules. The complete decolourisation process spanned 15 days, with each of the three active capsules receiving five days of treatment. The third and fourth chambers were maintained under identical conditions to the second, and sequential circulation facilitated the collection of clear, decolourised water from the final chamber. Prior to environmental release, the decolourised water was sterilised using the wet-heat sterilisation method of autoclaving to mitigate any potential ecological impact of *L. pseudotheobromae*. According to the operational process of the second-stage prototype, it was observed that the shaking speed should be reduced from 90 rpm to 50 rpm, as this facilitates proper mycelial growth in PDB.

The second-stage prototype demonstrated significant improvements in scalability, durability, and operational efficiency, representing an advancement in the application of fungal mycoremediation for synthetic dye removal in wastewater treatment. Decolourisation was initially assessed visually against a control; however, more precise quantification using advanced analytical techniques such as HPLC-MS and total organic carbon (TOC) analysis is planned. Although beyond the scope of the current study, future research will employ UPLC MS-MS facilities at Rajarata University of Sri Lanka to elucidate detailed degradation pathways and overall treatment efficiency.

At this stage, the focus was on evaluating the operational performance of the second-stage prototype. Subsequent studies will investigate a broader range of dye concentrations (0.01–0.2% w/v Crystal Violet) and incorporate advanced analytical techniques such as HPLC-MS and TOC testing to provide comprehensive and quantitative results.

## 5 Discussion

### 5.1 Synthetic dye pollution: environmental impact and the need for advanced treatment

The improper disposal and uncontrolled release of synthetic dyes from industries into aquatic environments are significant concerns contributing to environmental pollution in contemporary times (Li et al., 2021; Bharathi and Ga, 2022; Ganaie et al., 2023). Compounds such as CR, CV, MG, and Safranin, commonly utilised in laboratories (Islam et al., 2019; Bharathi and Ga, 2022; Shah et al., 2023), are often released without proper treatment. Such indiscriminate discharge poses a significant threat to the environment and human well-being (Li et al., 2021; Bharathi and Ga, 2022). Conventional physicochemical treatment methods for dye removal have proven largely ineffective in mitigating the environmental impacts and addressing the challenges, necessitating the development of more efficient treatment technologies (Yang et al., 2016; Routoula and Patwardhan, 2020; Ardila-Leal et al., 2021; Shah et al., 2023).

### 5.2 Enzymatic mechanisms in fungal biodegradation of synthetic dyes

The degradation of synthetic dyes involving fungi is mainly driven by biotransformation enzymes, which are crucial for breaking down complex dye molecules (Shi et al., 2021; Ngo and Tischler, 2022). The biosorption mechanism is recognised as an important factor in dye decolourisation by living fungi, contributing to the potential recovery of synthetic chemicals from spent dye baths (Fu and Viraraghavan, 2001; Khan et al., 2013). Mycoremediation has emerged as a prominent alternative in addressing synthetic dye pollution. Fungi, with their distinctive characteristics, play a crucial role in the remediation process, making them essential contributors to mitigating the environmental issues associated with synthetic dyes (Yang et al., 2016; Chen et al., 2019; Melati et al., 2022; Shourie and Vijayalakshmi, 2022; Thoa et al., 2023; Vaksmaa et al., 2023). The ability of fungi to degrade pollutants is attributed to their extracellular enzyme system, which is non-specific and non-stereoselective (Hofrichter, 2002; Khan et al., 2013). Fungal species capable of producing ligninolytic enzymes, including laccase, manganese peroxidase (MnP), and lignin peroxidase (LiP), have been widely used in treating resilient synthetic dyes in wastewaters (Hofrichter, 2002; Torres-Farradá et al., 2017; Dayi et al., 2018; Borham et al., 2023). These enzymes, derived from lignolytic fungi, have demonstrated significant effectiveness in decolourising dyes in textile wastewater systems (Khan et al., 2013; Daphedar et al., 2023).

Laccase, along with immobilised laccases, has been extensively investigated for its role in degrading recalcitrant compounds, including synthetic dyes (Abadulla et al., 2000; Saratale et al., 2009). Fungi can transform dyes, particularly azo dyes, into cation radicals, making them susceptible to nucleophilic attacks by water or hydrogen peroxide. This transformation leads to both symmetrical and asymmetrical cleavage of the azo bonds, generating intermediate compounds that undergo multiple redox reactions before stabilising into less toxic forms (Khan et al., 2013). Unlike direct cleavage of azo bonds, laccase-mediated decolourisation operates through a highly non-specific free radical mechanism, preventing the formation of toxic aromatic amines (Kalme et al., 2009). Furthermore, MnP isoenzymes efficiently decolourize azo dyes and phthalocyanine complexes in a Mn^2+^-independent manner. This enzymatic treatment not only disrupts chromophoric groups but also significantly alters the overall chemical structure of the dyes, facilitating their breakdown (Chagas and Durrant, 2001; Khan et al., 2013).

In this study, the main contributors to synthetic dye decolourisation among the tested fungi were *Lasiodiplodia crassispora* and *L. pseudotheobromae*, particularly in the decolourisation of CV. Of these two species, *L. pseudotheobromae* was selected for prototype application due to its superior performance in CV decolourisation. Considering its strong dye-decolourising ability, the genus *Lasiodiplodia* is recognised for producing ligninolytic enzymes, especially laccase (Esteves et al., 2014; Salvatore et al., 2020; Borham et al., 2023).

In our studies, *L. pseudotheobromae* demonstrated laccase enzyme production, as identified through qualitative enzymatic assays. Laccase activity was further assessed using application of 0.1 M 1-Naphthol (Research Lab Fine Chem. Industries, Mumbai, India) on five-day-old cultures of *L. pseudotheobromae*. After reagent application, plates were incubated for an additional 24 hours at 28–30 °C. A visible colour change at the colony edge, particularly the development of a blue–purple colouration, was considered a positive indication of laccase activity, following the methods described by Robledo-Mahón et al. (2020). The laccase-producing ability of the same *L. pseudotheobromae* strain used in this study was also confirmed by Wimalasena et al. (2024).

### 5.3 Previous evidence of dye degradation efficiency of *Lasiodiplodia* spp. and *Aspergillus* spp.

*Lasiodiplodia* sp. belongs to the family *Botryosphaeriaceae* (Jami et al., 2013; Wijayawardene et al., 2022), and is identified as an endophytic fungus living within the host plant tissues (Borham et al., 2023). *Lasiodiplodia* sp. has been identified as a producer of lignocellulolytic enzymes, with a focus on laccase and MnP, showcasing potential applications in mycoremediation against diverse synthetic dye contaminants (Arunprasath et al., 2019; Borham et al., 2023). Additionally, Félix et al. (2018) documented the ability of *Lasiodiplodia* strains to produce various extracellular enzymes, including cellulases, lipases, xylanases, and pectinases.

Considering previous studies, Borham et al. (2023) documented a remarkable 94.8% decolourisation ability of *Lasiodiplodia* sp. for 250 mg L^-1^ of CR, while Asses et al. (2018) reported a higher decolourisation efficiency of 97% for CR (200 mg L^-1^) by the same fungal species. Besides, Arunprasath et al. (2019) highlighted the mycelium of *Lasiodiplodia* sp. and its role in the adsorption of MG, achieving an impressive decolourisation efficiency of 96.9%. Nguyen et al. (2017) reported CV decolourisation ability of *Lasiodiplodia* sp. was 93.2% (10 mg L^-1^). Likewise, this study also provided significant evidences of the synthetic dye decolourisation ability of *Lasiodiplodia* sp. in CV, CR, and MG.

Nevertheless*, Aspergillus* sp. has been utilised for dye removal through processes such as absorption by mycelium, degradation with ligninolytic enzymes, or a combination of both, as outlined by Liu et al. (2020). Namnuch et al. (2021) reported the capability of *Aspergillus* sp. to produce cellulolytic enzymes. Ekanayake and Manage (2022) highlighted that *A. niger* could produce laccase enzymes, directly contributing to textile dye decolourisation. Other previous studies have demonstrated the significant contribution of *Aspergillus* sp. in synthetic dye decolourisation. For instance, the isolated fungus *A. flavus* exhibited the ability to achieve a substantial reduction of synthetic dyes, including CV, MG, and safranin, ranging from 80% to 90% within a one-week period (Lalitha et al., 2011). Furthermore, Asses et al. (2018) reported a 97% decolourisation of CR (200 mg L^-1^) by *A. niger*, and Skanda et al. (2021) found that *A. arcoverdensis* achieved an impressive 98.6% decolourisation of CR (100 mg L^-1^).

### 5.4 Efficiency of tested fungi in dye decolourisation: *in vitro* screening

In this study, we investigated the potential efficacy of fungi isolated from Mahakanadara and Mihintale tanks in the Anuradhapura district of Sri Lanka for the remediation of synthetic dyes. Specifically, *Aspergillus* sp., *Lasiodiplodia crassispora*, *L. pseudotheobromae*, *Neopestalotiopsis saprophytica*, and *Trichoderma* sp. were evaluated for their ability to decolourize CR, CV, MG, and Safranin through *in vitro* screening. The selected fungal strains demonstrated robust growth, reaching a colony diameter of 6.5 cm on PDA within one week. Their rapid growth rate minimised the risk of contamination by other fungi and bacteria. Furthermore, these fungi exhibited adaptability to both solid and liquid PDA media, underscoring their resilience under different culture conditions. Synthetic dyes, commonly discharged from laboratories and textile industries without adequate treatment, pose a significant environmental hazard. The *in vitro* assay conducted in this study revealed substantial decolourisation potential among the selected fungal strains, with *Aspergillus* sp., *L. crassispora*, and *L. pseudotheobromae* exhibiting the most pronounced effects. Notably, *L. crassispora* demonstrated the highest decolourisation efficiency, surpassing 70% for all tested dyes. These findings highlight the potential application of *Lasiodiplodia* sp. as a bioremediation agent for synthetic dye removal, offering an effective strategy for mitigating environmental pollution.

### 5.5 Efficiency of tested fungi in dye decolourisation: prototype development

Beyond laboratory-scale experimentation, these findings hold significant potential for industrial applications. The prototype was developed in two progressive stages to bridge the gap between research and large-scale implementation. The initial prototype successfully revealed the feasibility of fungal-based decolourisation of synthetic dyes through a stepwise, gravity-driven system. The integration of *Lasiodiplodia pseudotheobromae* within sequential treatment chambers effectively facilitated dye degradation, with CV achieving complete decolourisation within four days, surpassing initial expectations. The reliance on passive water flow and controlled agitation further enhanced the system’s sustainability, offering an eco-friendly approach to wastewater treatment.

Despite its success, the first-stage prototype exhibited limitations in water storage capacity, sterilization compatibility, and scalability, necessitating design refinements for industrial applicability. In response, a second-stage prototype was engineered, incorporating larger glass treatment chambers, an electronically controlled shaking mechanism, and a water pumping system to enable continuous treatment. These modifications significantly improved the system’s efficiency, operational durability, and scalability, making it more suitable for large-scale wastewater remediation.

The transition from the initial to the advanced prototype highlights the viability of fungal mycoremediation as a sustainable and efficient strategy for synthetic dye removal. Future optimizations and pilot-scale evaluations will be critical to refining the system for seamless industrial integration, ensuring its practical applicability in large-scale wastewater treatment operations.

### 5.6 Cost analysis of second-stage prototype vs. conventional dye decolourisation methods

According to our experience, the total cost of preparing the second-stage prototype was approximately 900 USD, which comprised raw materials and consumables (USD 399), equipment and tools (USD 166), prototype development and fabrication (USD 166), labour costs (USD 116), and logistics and maintenance (USD 33). This prototype effectively decolourised 100 ppm CV using *Lasiodiplodia pseudotheobromae* within 15 days. The cost figure reflects only the development expenses; subsequent costs are limited to maintenance and the recurrent expense of PDB. Overall, this design is eco-friendly, cost-effective, and durable, with a projected usable lifespan exceeding one year.

Saad et al. (2022) reported that the Malaysian textile industry generates azo dye wastewater, which is difficult to treat biologically due to its chemical stability. Conventional treatment methods, such as electrocoagulation (EC) and photocatalysis, are often costly and energy-intensive. In their study, both standalone EC and integrated electrocoagulation–membrane (ECM) systems achieved a 96% dye removal efficiency. Cost analysis revealed that the ECM system (1 V, 1.0 g NaCl) incurred a total cost of 1.079 million MYR (approximately USD 256,800), whereas the standalone EC system (1 V, 1.0 g NaCl) was even more cost-effective, with a total cost of 0.325 million MYR (approximately USD 77,000).

### 5.7 Limitations and recommendations

This study highlights the potential of fungal strains in synthetic dye degradation; however, certain limitations should be recognised. Only a single dye concentration of 0.01% (w/v) was tested in both solid and liquid media. While this concentration was selected as a standard baseline commonly reported in fungal dye decolourisation studies, it may not be sufficient to fully evaluate the tolerance and degradation capacity of the fungi investigated. Future studies should therefore employ a broader concentration gradient (0.01–0.2% w/v) to provide a more comprehensive understanding and improve the reliability of results.

In addition, the observed colour removal does not necessarily indicate complete mineralisation of the dye, as the assessment was based solely on visual decolourisation relative to the control. More rigorous evaluation requires advanced analytical techniques such as HPLC-MS and total organic carbon (TOC) testing. Although beyond the scope of the present study, future research will utilise UPLC MS-MS facilities at Rajarata University of Sri Lanka to gain deeper insights into the degradation pathways and overall efficiency.

### 5.8 The role of commonly found fungi in various remediation fields as future directions

This section highlights the importance of commonly encountered fungi and their potential applications in various remediation fields. It stresses the need to go beyond fungal isolation and identification to explore their broader roles in bioremediation. The fungi examined in this study, including *Aspergillus* spp., *Lasiodiplodia* spp., *Neopestalotiopsis* spp., and *Trichoderma* spp., are frequently found in diverse environments, often as plant pathogens or laboratory contaminants.

Among these, *Lasiodiplodia theobromae* is a well-known plant pathogen prevalent in tropical and subtropical regions (Salvatore et al., 2020). Similarly, species within *Pestalotiopsis* are commonly reported as saprobic fungi (Maharachchikumbura et al., 2014; Belisário et al., 2020). *Aspergillus* spp., *Alternaria* spp., and *Penicillium* spp. are frequently observed as laboratory contaminants (Leifert and Woodward, 1998). Despite their ubiquity and potential for contamination, these fungi possess significant yet often overlooked roles in mycoremediation. Recognizing their bioremediation potential is crucial for maximizing their positive environmental impact.

Kang et al. (2019) reported that *Neopestalotiopsis* spp. are involved in the degradation of pharmaceutical compounds, while *Lasiodiplodia* spp. have demonstrated efficiency in heavy metal degradation (Deng et al., 2014) and textile dye degradation (Borham et al., 2023). Among these fungi, *Trichoderma* spp. are well-documented for their bioremediation capabilities, particularly in degrading various pollutants (Ren et al., 2022; Racić et al., 2023; Voloshchuk et al., 2024; Conte et al., 2025). *Trichoderma harzianum* (AUMC14897) has also been reported for efficient dye decolourisation in wastewater treatment through laccase enzyme production, effectively degrading Novatic Green XBN, Red 4BL, and Reactive T. Blue G (Salem et al., 2024). Furthermore, *T. viride* exhibited decolourisation efficiencies of 36%, 73%, and 87% for Synozol Red, Yellow, and Navy Blue dyes, respectively (Ali et al., 2023). Al-Hawash et al. (2018) reported that *T. harzianum* demonstrates significant hydrocarbon degradation potential, mainly through laccase enzyme production.

Similarly, *Aspergillus* spp. play a significant role in bioremediation processes (El-Bondkly and El-Gendy, 2022; Zango et al., 2023; Ghanaim et al., 2025; Ameen et al., 2024). These species serve as prime examples of fungi with substantial remediation potential. The role of *Aspergillus* spp. in synthetic dye decolourisation has also been well documented. For instance, *A. fumigatus* and *A. flavus* were found to degrade Sumifex Turquoise Blue and Navy Blue dyes (Ghafoor et al., 2024). *Aspergillus candidus* decolourised Direct Blue 71 (Rathi and Kumar, 2022), while *A. niger*, *A. candidus*, and *A. iizukae* were effective in decolourizing Direct Yellow (Kořínková et al., 2019). Additionally, *A. fumigatus* exhibited degradation potential for Rhodamine B (Vithalani and Bhatt, 2023), and *A. tamarii* demonstrated a 97.87% decolourisation efficiency for Remazol Black B (Ayu and Kasiamdari, 2022

The role of fungi in petroleum hydrocarbon remediation is another significant area of study. Al-Hawash et al. (2018) reported that *A. fumigatus*, *A. ochraceus*, *A. niger*, and *T. harzianum* exhibit remarkable hydrocarbon degradation potential, primarily through the production of extracellular enzymes. *Aspergillus niger* demonstrated a hydrocarbon degradation efficiency of 94.4%, while *P. commune* displayed a degradation activity ranging from 80% to 90% (Myazin et al., 2021; Mahmud et al., 2022).

Fungal involvement in heavy metal remediation has been extensively studied. Several fungi and yeast strains, including *Aspergillus* spp., *Candida* spp., *Fusarium* spp., *Mucor* spp., *Penicillium* spp., and *Saccharomyces* spp., have been reported to effectively remediate heavy metals such as Cd, Cr, Cu, Ni, Co, Pb, and Zn in contaminated soils (Archana and Jaitly, 2015; Siddiquee et al., 2015; Kumar and Bharadvaja, 2020; Kumar and Dwivedi, 2021).

## 6 Conclusion

*In-vitro* screening aimed at assessing the decolourisation potential of by filamentous ascomycetous fungi isolated from freshwater environments in Sri Lanka for synthetic dyes (CR, CV, MG, and Safranine), both solid and liquid media states, were employed. Visual observations revealed that CV was decolourised by all isolated fungal species, while CR was effectively decolourised by *Lasiodiplodia crassispora, L. pseudotheobromae*, and *Aspergillus* sp., with *Neopestalotiopsis saprophytica* and *Trichoderma* sp. exhibiting no decolourisation ability for CR. Efficient decolourisation of MG was observed with *L. crassispora* and *L. pseudotheobromae*, and Safranin was effectively decolourised by *L. crassispora*. In the liquid media assay, absorbance measurements after 14 and 28 days of fungal inoculation represented crucial indicators of dye remediation efficacy. Robust decolourisation performance, exceeding 85%, was achieved by *Aspergillus* sp., *L. crassispora, L. pseudotheobromae*, and *N. saprophytica* for CV after 14 days. Similarly, superior decolourisation, surpassing 85%, was demonstrated by *Aspergillus* sp., *L. crassispora*, and *L. pseudotheobromae* for CR. Notably, *L. crassispora* and *L. pseudotheobromae* reported remarkable efficacy, with over 85% decolourisation in MG.

Based on assays conducted in both solid and liquid media, *L. pseudotheobromae* was selected for prototype application owing to its superior ability to decolourise CV compared with the other fungal isolates tested. Crystal Violet was chosen for the prototype as it demonstrated the most rapid and consistent decolourisation among the dyes evaluated. Accordingly, the prototype was developed in two stages. The first-stage prototype had certain limitations, including low water storage capacity, which restricted continuous filtration and durability. In addition, the use of an orbital shaker, while effective at small scale, proved impractical for larger-scale applications, and the overall process efficiency was limited. The second-stage prototype successfully addressed these limitations, achieving complete decolourisation within 15 days under optimised conditions of 90 rpm shaking speed, 30 °C incubation temperature, and average pH 6.5. The design is eco-friendly, cost-effective, and durable, with strong potential for implementation in large-scale industrial applications for the decolourisation of wastewater contaminated with synthetic dyes.

## Data Availability

The datasets presented in this study can be found in online repositories. The names of the repository/repositories and accession number(s) can be found in the article/**Supplementary Material**.

## Supplementary material

Supplementary Figure 1 Screening of the CV dye decolourisation ability of the fungi in liquid media after 14 days of inoculation period. (C_1_) Negative control - CV, **(A)** *Lasiodiplodia crassispora*, **(B)** *L. pseudotheobromae*, **(C)** *Neopestalotiopsis saprophytica*, **(D)** *Aspergillus* sp., and **(E)** *Trichoderma* sp. **(A–E)** Positive control.

Supplementary Figure 2 Screening of the CR decolourisation ability of the fungi in liquid media after 14 days of inoculation. (C_2_) Negative control - CR, **(A)** *Lasiodiplodia crassispora*, **(B)** *L. pseudotheobromae*, **(C)** *Neopestalotiopsis saprophytica*, **(D)** *Aspergillus* sp., and **(E)** *Trichoderma* sp. **(A–E)** Positive control.

Supplementary Figure 3 Screening of the safranin decolourisation ability of the fungi in liquid media after 14 days of inoculation. (C_3_) Negative control – Safranin, **(A)** *Lasiodiplodia crassispora*, **(B)** *L. pseudotheobromae*, **(C)** *Neopestalotiopsis saprophytica*, **(D)** *Aspergillus* sp. and **(E)** *Trichoderma* sp. **(A–E)** Positive control.

Supplementary Figure 4 Screening of the MG decolourisation ability of the fungi in liquid media after 14 days of inoculation. (C_4_) Negative control - MG, **(A)** *Lasiodiplodia crassispora*, **(B)** *L. pseudotheobromae*, **(C)** *Neopestalotiopsis saprophytica*, **(D)** *Aspergillus* sp., and **(E)** *Trichoderma* sp. **(A–E)** Positive control.
