# Supplementary material for: In-vitro evaluation of synthetic dye decolourisation by filamentous ascomycetous fungi isolated from freshwater environments in Sri Lanka and development of a prototype for addressing environmental pollution from synthetic dye contamination
Source: Front Cell Infect Microbiol. 2025 Oct 23;15:1650835. doi: 10.3389/fcimb.2025.1650835 (PMC12589041; doi:10.3389/fcimb.2025.1650835)
Supplement: Supplementary file 1 [file Table1.docx]

Supplementary Material

# Supplementary Data

**Table 1:** Details of sequences used for *Lasiodiplodia crassispora* (RUFCC2463) and *L. pseudotheobromae* (RUFCC2464) phylogenetic analyses

| Species | Voucher/Strain | GenBank accession numbers | |
| --- | --- | --- | --- |
|  |  | ITS | *tef*1-α |
| *Lasiodiplodia crassispora* | **RUFCC2463** | **PQ327547** | **PQ336773** |
| *Lasiodiplodia pseudotheobromae* | **RUFCC2464** | **PQ327548** | N/A |

Note: Rajarata University Fungal Culture Collection (RUFCC)
