# Supplementary figures and images for: In-vitro evaluation of synthetic dye decolourisation by filamentous ascomycetous fungi isolated from freshwater environments in Sri Lanka and development of a prototype for addressing environmental pollution from synthetic dye contamination
Source: Front Cell Infect Microbiol. 2025 Oct 23;15:1650835. doi: 10.3389/fcimb.2025.1650835 (PMC12589041; doi:10.3389/fcimb.2025.1650835)

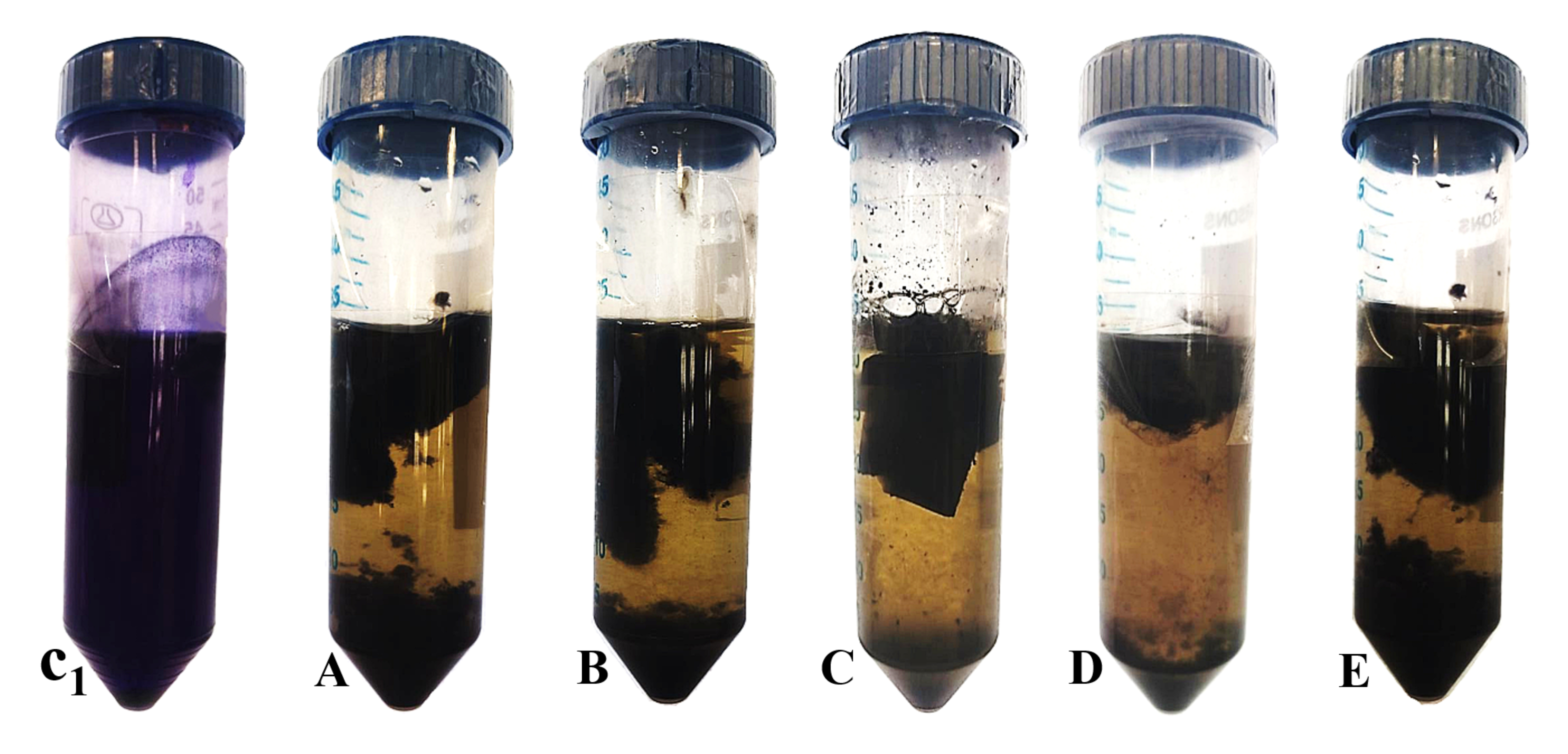

Supplement: Supplementary Figure 1 — Screening of the CV dye decolourisation ability of the fungi in liquid media after 14 days of inoculation period. (C1) Negative control - CV, (A) Lasiodiplodia crassispora, (B) L. pseudotheobromae, (C) Neopestalotiopsis saprophytica, (D) Aspergillus sp., and (E) Trichoderma sp. (A–E) Positive control. [file Image1.jpeg]

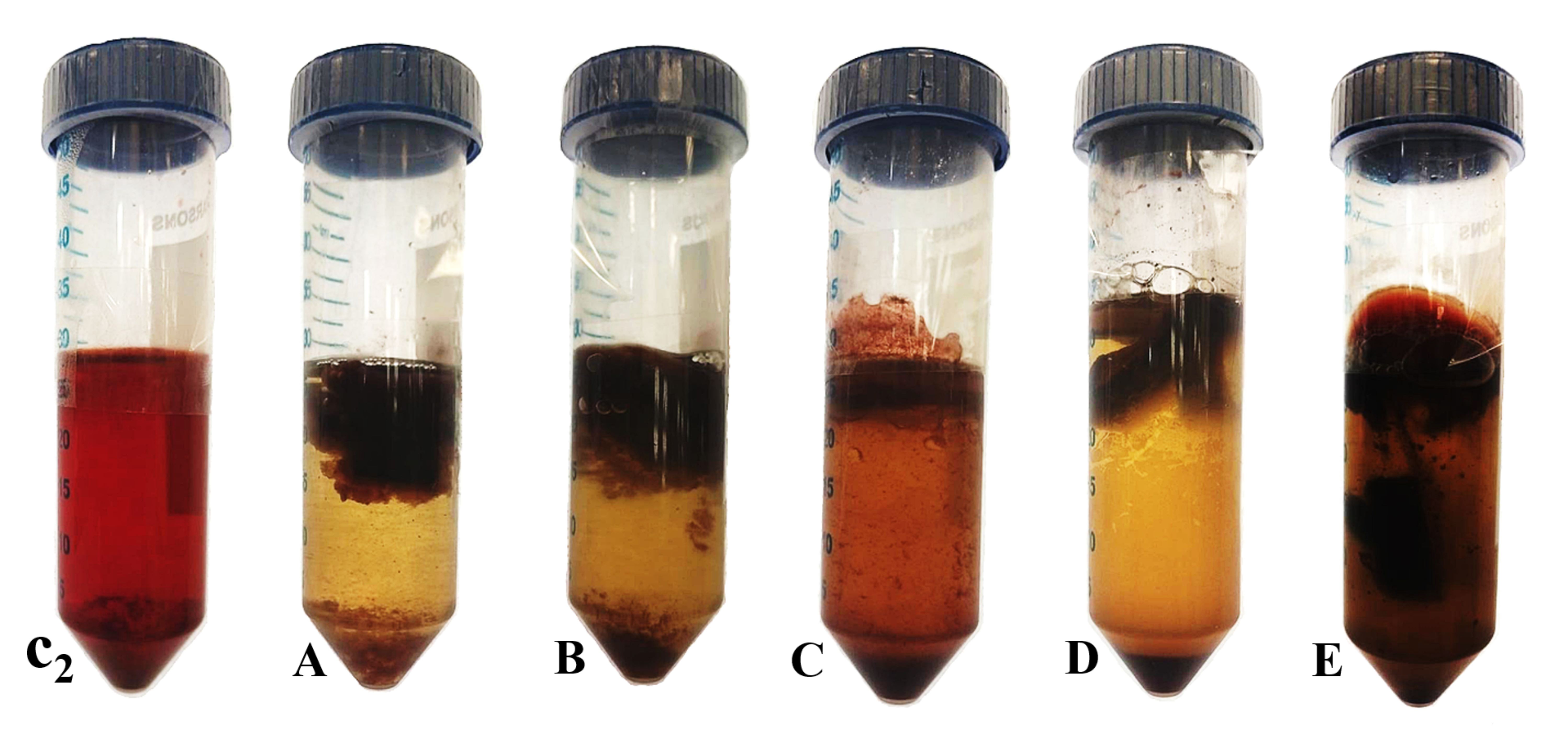

Supplement: Supplementary Figure 2 — Screening of the CR decolourisation ability of the fungi in liquid media after 14 days of inoculation. (C2) Negative control - CR, (A) Lasiodiplodia crassispora, (B) L. pseudotheobromae, (C) Neopestalotiopsis saprophytica, (D) Aspergillus sp., and (E) Trichoderma sp. (A–E) Positive control. [file Image2.jpeg]

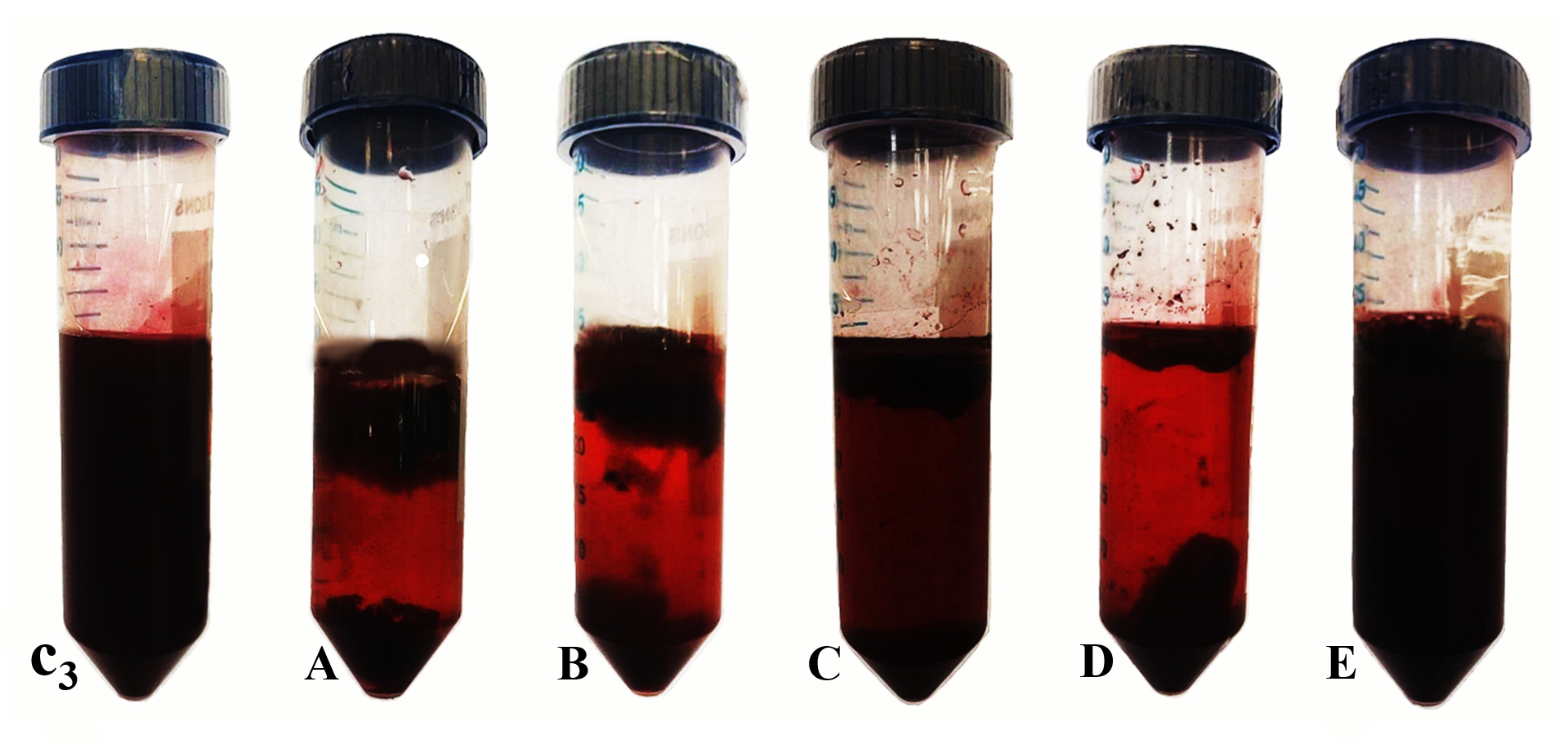

Supplement: Supplementary Figure 3 — Screening of the safranin decolourisation ability of the fungi in liquid media after 14 days of inoculation. (C3) Negative control – Safranin, (A) Lasiodiplodia crassispora, (B) L. pseudotheobromae, (C) Neopestalotiopsis saprophytica, (D) Aspergillus sp. and (E) Trichoderma sp. (A–E) Positive control. [file Image3.jpeg]

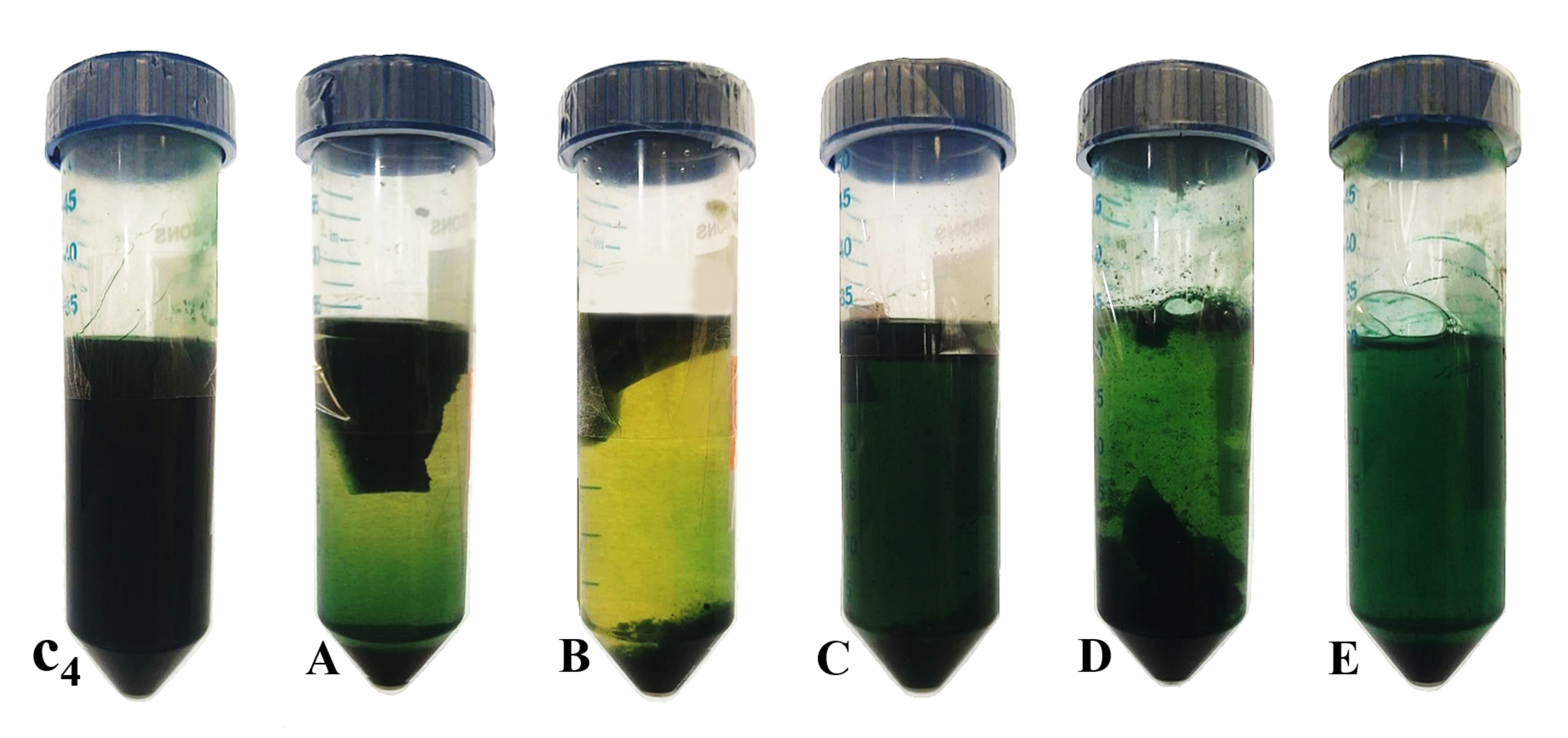

Supplement: Supplementary Figure 4 — Screening of the MG decolourisation ability of the fungi in liquid media after 14 days of inoculation. (C4) Negative control - MG, (A) Lasiodiplodia crassispora, (B) L. pseudotheobromae, (C) Neopestalotiopsis saprophytica, (D) Aspergillus sp., and (E) Trichoderma sp. (A–E) Positive control. [file Image4.jpeg]
